# Simplified two-compartment neuron with calcium dynamics capturing brain-state specific apical-amplification, -isolation and -drive

**DOI:** 10.3389/fncom.2025.1566196

**Published:** 2025-05-20

**Authors:** Elena Pastorelli, Alper Yegenoglu, Nicole Kolodziej, Willem Wybo, Francesco Simula, Sandra Diaz-Pier, Johan Frederik Storm, Pier Stanislao Paolucci

**Affiliations:** ^1^Istituto Nazionale di Fisica Nucleare, Sezione di Roma, Rome, Italy; ^2^Simulation and Data Lab Neuroscience, Jülich Supercomputing Centre (JSC), Institute for Advanced Simulation, JARA, Jülich Research Center, Jülich, Germany; ^3^Department of Mathematics, Institute of Geometry and Applied Mathematics, RWTH Aachen University, Aachen, Germany; ^4^Dipartimento di Fisica, Università di Roma Sapienza, Rome, Italy; ^5^Institute of Mediterranean Neurobiology (INMED), Institut National de la Santé et de la Recherche Médicale (INSERM), Turing Centre for Living Systems (CENTURI), Aix-Marseille Université, Marseille, France; ^6^Peter Grünberg Institute 15 – Neuromorphic Software Ecosystems, Jülich Research Center, Jülich, Germany; ^7^Brain Signaling Group, Section for Physiology, Department of Molecular Medicine, Institute of Basic Medical Sciences, University of Oslo, Oslo, Norway

**Keywords:** two-compartment neuron model, spiking networks, apical mechanisms, brain-states, multi-compartment neuron model, evolutionary algorithm, learning, sleep

## Abstract

Mounting experimental evidence suggests the hypothesis that brain-state-specific neural mechanisms, supported by the connectome shaped by evolution, could play a crucial role in integrating past and contextual knowledge with the current, incoming flow of evidence (e.g., from sensory systems). These mechanisms would operate across multiple spatial and temporal scales, necessitating dedicated support at the levels of individual neurons and synapses. A notable feature within the neocortex is the structure of large, deep pyramidal neurons, which exhibit a distinctive separation between an apical dendritic compartment and a basal dendritic/perisomatic compartment. This separation is characterized by distinct patterns of incoming connections and three brain-state-specific activation mechanisms, namely, apical-amplification, -isolation, and drive, which have been proposed to be associated - with wakefulness, deeper NREM sleep stages, and REM sleep, respectively. The cognitive roles of apical mechanisms have been supported by experiments in behaving animals. In contrast, classical models of learning in spiking networks are based on single-compartment neurons, lacking the ability to describe the integration of apical and basal/somatic information. This work provides the computational community with a two-compartment spiking neuron model that supports the proposed forms of brain-state-specific activity. A machine learning evolutionary algorithm, guided by a set of fitness functions, selected parameters defining neurons that express the desired apical dendritic mechanisms. The resulting spiking model can be further approximated by a piece-wise linear transfer function (ThetaPlanes) for use in large-scale bio-inspired artificial intelligence systems.

## 1 Introduction

**Figure 1:**
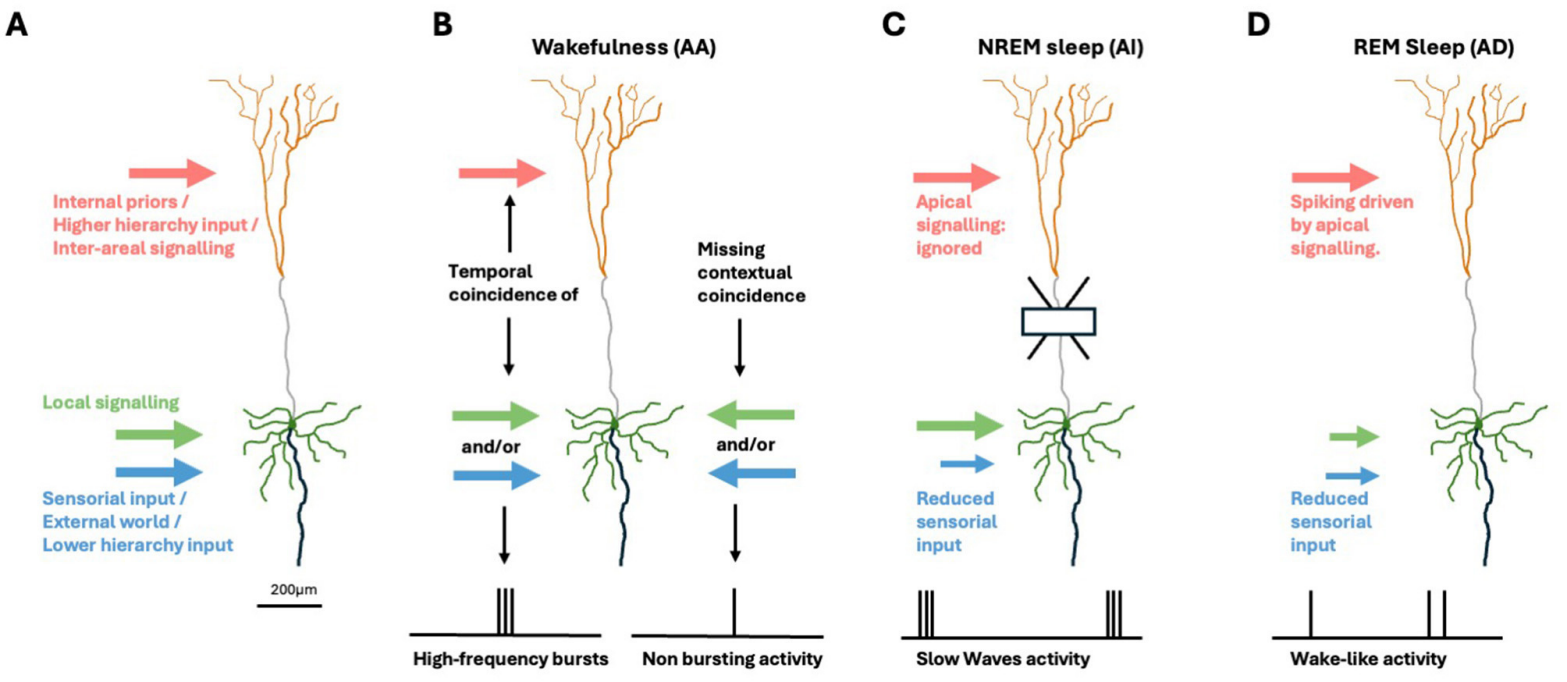
Brain-state specific apical mechanisms in pyramidal neurons. **(A)** Cortical pyramidal cell. Green: soma and (peri)somatic dendrites. Orange: apical dendritic tuft. Gray: apical dendrite. Black: axon. Inputs from other areas, representing internally generated priors and top-down signals from areas higher in the hierarchy are segregated to touch the apical tuft (light red arrow). Sensorial evidence and input from areas at lower hierarchical levels (light blue arrow) target the somatic/perisomatic zone together with local signaling (green arrow). **(B)** During wakefulness, the AA mechanism signals the temporal coincidence of (peri-)somatic and apical inputs by emitting high-frequency burst. **(C)** In deep-sleep, AI induces the soma to ignore apical signals. **(D)** When dreaming, AD induces a multi-areal integration driven by internal imagery in absence of sensorial inputs.

Thanks to an evolutionary history spanning hundreds of millions of years and selecting from countless individuals, the structural connectome and cellular mechanisms have become adept at supporting the integration of multi-modal sensory evidence with internal hypotheses about the world and the self (Papale et al., 2023; Muckli et al., 2015; Sporns et al., 2005; Hagmann et al., 2008). Additionally, specialized solutions have emerged at the macro-, meso-, and micro-scales, enabling the expression of dynamic repertoires of functional connectivity (Cabral et al., 2017). At the cellular level, within at least some types of large, cortical pyramidal cells of the mammalian neo-cortex (in layer 5: L5 PCs, see Figure 1), specific feed-forward sensory input can be amplified by contextual and feed-back information by the so-called *apical-amplification* (AA) principle (Phillips et al., 2016). In particular, Phillips (2023) and Phillips et al. (2025), building on previous studies (Larkum et al., 1999, 2009; Larkum, 2013), have proposed that AA and related apical dendritic mechanisms are among the cellular foundations of mental life. AA is thought to play an important role during wakefulness (Larkum, 2013; Phillips et al., 2016), by inducing high-frequency bursts in a subset of neurons that detect temporal coincidences between internal priors and the flow of external information. This makes them ideal candidates for the neural correlate of conscious perception and supports advantageous cognitive functions, including faster classification/recognition and more rapid, reliable learning of new patterns.

Evidence suggests that AA is replaced by distinct principles and mechanisms during transitions to other brain states (Aru et al., 2020a,b)—specifically, *apical isolation* (AI) during the deepest stages of NREM sleep (as in anesthesia; Suzuki and Larkum, 2020), and *apical drive* (AD) during REM sleep dreaming (Aru et al., 2020b)—and possibly during daydreaming, when attention to sensory inputs is reduced. The roles of AI and AD can be related to the functions of deep sleep and (day-)dreaming, respectively. Sleep has evolved and been maintained across all animal species studied, despite its apparent lack of productivity. There is increasing evidence that it promotes memory consolidation and integration, as well as preparation for anticipated tasks (Tononi and Cirelli, 2014; Buzsáki, 2015; Sejnowski and Destexhe, 2000), and returns the network to optimal functional state after periods of awake learning (Watson et al., 2016; Tononi and Cirelli, 2020). Mammals devote a significant portion of their time to sleep, especially at young age, when the overall learning rate is highest (Frank et al., 2001), whereas sleep deprivation negatively impacts cognitive performance (Killgore, 2010). These considerations underscore the importance of adequate modeling of sleep, including the underlying cellular mechanisms, and their impact on cognitive functions. In deep sleep, AI induces neurons to ignore inter-areal signaling, enabling local optimizations of the synaptic matrix, e.g., homeostasis and associations, with beneficial cognitive and energetic effects during post-sleep wakefulness like those demonstrated in Capone et al. (2019) and Golosio et al. (2021).

Here, we propose a method to transition from the classical modeling approach of networks, which relies on single-compartment *point-like* spiking neurons, toward incorporating simple apical dendritic mechanisms, in particular dendritic Ca2+-dynamics, specifically in an exemplary model configuration we named *Ca-AdEx*. This inclusion supports the expression of intriguing brain-state-specific learning capabilities. Also, it enables more efficient simulations to investigate the possible impact of dendritic mechanisms on conscious processing in the cortex, as proposed by the Dendritic Integration Theory (Aru et al., 2020a). Recently, Storm et al. (2024) have proposed that such subcellular mechanisms may provide a unifying principle that may help bridging gaps between different theories of consciousness, toward a more integrative, multi-scale view.

Single-compartment models with spike frequency adaptation, such as the Adaptive Exponential Integrate and Fire neuron (AdEx) (Brette and Gerstner, 2005), have enabled the construction of networks capable of entering both wakefulness-like asynchronous irregular regimes and deep-sleep-like synchronous slow oscillation regimes (e.g., Pastorelli et al., 2019; Capone et al., 2017). For such networks, mean-field models have been developed (di Volo et al., 2019). These mean-field descriptions of the behavior of spiking networks composed of AdEx neurons have supported the development of models based on connectomes at the scale of the whole mammalian/human brain (di Volo et al., 2019; Aquilué-Llorens et al., 2023), also capable of expressing both the asynchronous and synchronous regimes. However, these models do not capture the activity of individual neurons and synapses in engram coding, nor do they support the simulation of the temporal evolution of engrams (Josselyn et al., 2015).

Targeting the simulation of intrinsic and network rhythmogenesis of the CA3 region (Pinsky and Rinzel, 1994) have introduced a two-compartment model of hippocampal pyramidal neurons. Even if this model includes a superset of the intrinsic currents of the distal compartment of Ca-AdEx, it is missing what needed to detect the temporal coincidence of apical and (peri-)somatic signals, later experimentally demonstrated by Larkum et al. (1999), and does not consider the later AD and AI hypotheses. Notably, we have used as starting point for the distal compartment of the novel Ca-AdEx the (Larkum et al., 2004) model, that demonstrates how a dendritic input can increase the gain of layer 5 pyramidal neurons, to which we have added the Ca2+dependent K current (essential to terminate Ca2+spikes), a more accurate modeling of the somatic compartment, and what necessary to address the emerging AI and AD experimental hypotheses. In a seminal study of the effect of Sleep Slow Oscillations on memory consolidation, Wei et al. (2016) have proposed a two-compartment model that, also including a similar set of intrinsic current, have been conceived to simulate only deep sleep oscillations, therefore not targeting wakeful AA and dreaming AD mechanisms.

The cognitive and energetic functions specific to different brain states, enabled by AA and AI mechanism, have been explored in spiking models engaged in learning and sleep cycles. These models aim to simulate the activity and contribution of individual neurons and monitor synaptic changes over time (Capone et al., 2019; Golosio et al., 2021; De Luca et al., 2023). Although these models utilize the temporal coincidence between contextual and perceptual information emulating AA mechanism during wakeful learning, and AI like mechanisms during deep-sleep, they are still based on single-compartment neurons. Therefore, they necessitate precise calibration of currents carrying contextual priors and novel evidence. Such modeling approaches cannot fully leverage the capabilities of apical mechanisms, for example, the transition to much higher frequencies associated with *apical-amplification* during wakefulness, *apical-drive* during dreaming, or *apical-isolation* during deep, slow-wave sleep.

Within the framework of bio-inspired artificial intelligence, a few studies (e.g., Capone et al., 2023b, 2022) have begun to explore the specific advantages of apical-amplification-like bursting mechanisms for fast learning in spiking networks engaged in complex temporal tasks. However, these models have taken as working hypotheses the existence of transfer functions that enter a bursting regime when a temporal coincidence between perceptual and sensorial signals is detected. Here, we demonstrate how to construct a two-compartment neuron model based on cellular biophysical evidence, capable of supporting an apical-amplification bursting mechanism.

Furthermore, bio-inspired artificial intelligence algorithms would benefit from neural models characterized by a simple transfer function, simplifying the definition of training rules. A classic transfer function adopted in artificial intelligence is the ReLU (rectified linear unit) rule, which approximates the transfer function of single-compartment neurons. We will show how to introduce a transfer function suitable for approximating the response of the two-compartment neuron to the combination (*I*_*s*_, *I*_*d*_) of somatic and distal signals, capable of describing the apical-amplification, -isolation, and -drive regimes. We have named this transfer function *ThetaPlanes* (*I*_*s*_, *I*_*d*_).

The extension of the AdEx model to include an apical compartment with simplified Ca2+-dynamics (the Ca-hotzone, here abbreviated to Ca-HZ) requires a few tens of parameters, implying a search in a high-dimensional space for fine-tuning. For any mathematical model, understanding the sensitivity of the model output to perturbations and correlations among the parameters defining it is crucial. This need becomes even more apparent when dealing with high-dimensional parameter spaces, where the dependency of outputs on underlying parameters becomes less intuitive for the modeler. As in many other research fields, neuroscience demands a thorough understanding of these relationships to draw meaningful conclusions about the simulated behavior of the modeled phenomena (Nowke et al., 2018; Yegenoglu et al., 2022). Population-based optimization techniques offer a more efficient approach to exploring large parameter spaces than brute-force testing of all possible parameter combinations. Depending on the shape of the manifolds, different algorithms may be more or less effective in navigating the parameter space and identifying areas of interest to the modeler. While, for example, gradient-based methods typically identify local minima and converge very quickly, not all fitness evaluation measures and parameter spaces are suitable for such algorithms (Yegenoglu et al., 2020). Simulated annealing and cross-entropy methods provide suitable gradient-free exploration techniques but also require fine-tuning of hyperparameters. In contrast, evolutionary strategies and similar population-based methods can effectively navigate complex parameter spaces and quickly adapt to the manifolds if the level of noise or stochasticity is maintained at a suitable level, depending on the variations induced by the parameters with respect to the fitness. Several such algorithms can be tested and even combined to achieve a comprehensive understanding of parameter sensitivity and interdependencies. The tools and methodology adopted in this work to explore the parameter space defining the two-compartment model we named *Ca-AdEx* are detailed in dedicated Section 2.

Multi-compartment (MC) models have been successful in reproducing experimentally observed dendritic processes and computations (Segev, 2000), particularly the interaction between apical Ca2+-spikes and somatic action potentials (Larkum et al., 2004; Hay et al., 2011; Shai et al., 2015). Most often, MC models are paired with Hodgkin-Huxley (HH) type ionic channels. The spatially extended nature of the dendritic tree, requiring many compartments, leads to models that are expensive to simulate. Past simplification efforts have focused on two largely orthogonal axes of advance: either condensing the HH channels into a simpler effective spike generation mechanism (Kistler et al., 1997; Pozzorini et al., 2015) or reducing the number of compartments needed in a simulation while maintaining desired response properties (Wybo et al., 2021). To ultimately arrive at the most efficient formulation of a neuron model, a simplified description of dendritic non-linearities needs to be combined with a reduction in the number of compartments, in such a way that the model architecture is flexible and can admit a range of dendritic computations. Previous work on this topic used a hybrid combination of compartment dynamics and kernel convolutions (Naud et al., 2014), the former to model Ca2+-activation and the latter to capture the somato-dendritic interactions. While the use of convolutions is a general way to capture the linear component of intra-dendritic interactions (Wybo et al., 2013, 2015), it is computationally inefficient compared to the use of normal coupling terms between compartments (Wybo et al., 2021). For this reason, we propose an approach that solely relies on normal compartmental dynamics, which has the added advantage of potentially integrating any type of non-linear conductance. By design, this approach can thus also implement other dendritic non-linearities, such as N-Methyl-D-Aspartate (NMDA) spikes (Schiller et al., 2000; Major et al., 2008, 2013). We demonstrate this potential by extending the two-compartment Ca-AdEx model to a multi-compartment description, which, next to the Ca-HZ and soma compartments, features apical and basal compartments suited for NMDA-spike generation. Furthermore, we have implemented a compartmental modeling framework in NEST (Gewaltig and Diesmann, 2007; Spreizer et al., 2022) that supports the aforementioned Ca2+-, AdEx-, and NMDA dynamics. Combined, our work facilitates the study of dendritic dynamics with simplified neuron models at the network level.

## 2 Materials and methods

### 2.1 The two-compartment Ca-AdEx model supporting calcium spike firing

One of the focal points of this endeavor has been the creation of a neuron model able to express properties of apical-amplification during awake states, to aid with the formation of memories inside the synaptic matrix during incremental learning cycles. Also, recent studies (Aru et al., 2020a,b) have supported the idea that apical-amplification in layer 5 pyramidal neurons (L5PC) plays a critical role for conscious sensory processing during the awake state, in contrast to the mechanisms of apical-drive and apical-isolation that are predominant in REM and NREM sleep, respectively (Aru et al., 2020a). To replicate these states in a model, it is essential to have a Ca-HZ in the apical compartment that is able to support Ca2+spike, (Larkum et al., 1999; Larkum, 2013), which is assumed to be the cellular mechanism underpinning apical-amplification.

#### 2.1.1 Dynamics of the two-compartment Ca-AdEx neuron

**Figure 2:**
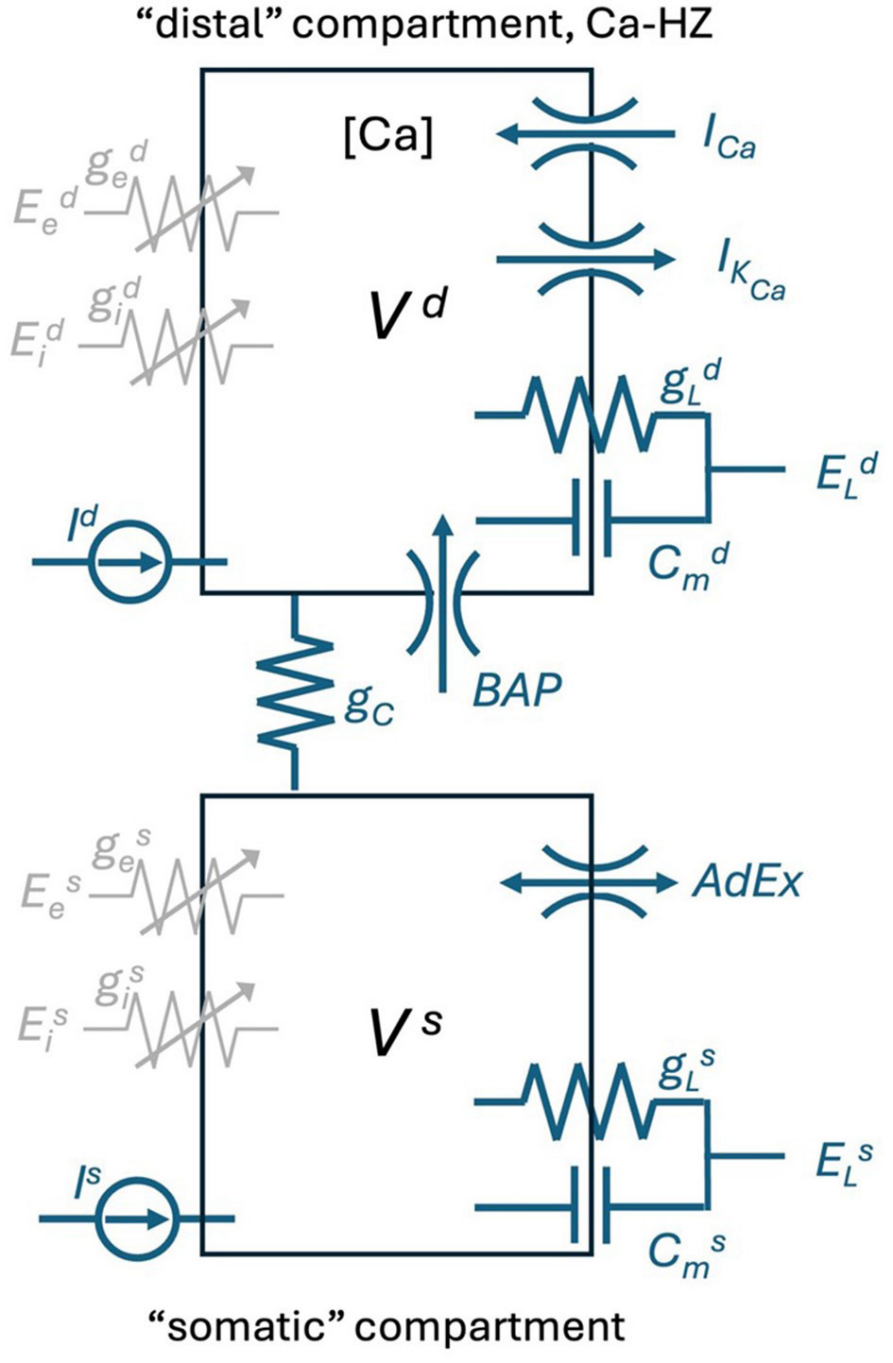
Schematic of the Ca-AdEx neuron. The model consists of two compartments: a basal compartment (i.e., somatic/perisomatic/basal dendritic) and a distal compartment (apical dendritic), coupled through a conductance *g*_*C*_ (roughly representing the axial conductance of the main apical dendritic trunk). In the distal compartment, the ionic channels for the *I*_*Ca*_ and *I*_*K*_*Ca*__ currents contribute to the Ca-HZ and to the dynamics of the calcium concentration [Ca]. The “Backpropagating Action Potential” from the somatic to the distal compartment is represented by the “BAP” channel. In the soma, the terms of the AdEx approximations in Equation 1 are represented by the “AdEx” channel. The *g*_*e*_ and *g*_*i*_ in each compartment represent ligand-gated ion channel conductances for excitatory/inhibitory input synapses. The membrane capacitance *C*_*m*_, the leakage conductance *g*_*L*_, the reversal potential *E*_*L*_, the input current *I*, and the membrane voltage *V* are indicated for each compartment. The model is also equipped with GABA, AMPA, NMDA, and AMPA+NMDA conductances and of a “synaptic input channel” for injecting a beta-shaped current (utilized in the *pulse stimuli* task to simulate EPSP-shaped input currents to the distal compartment), not reported in this schematic.

The inter-spike dynamics of the two compartment model (outside the refractory period, if specified) is summarized by the following equations:


(1) $$\left\{ \begin{array}{l} \left\{ \begin{array}{l} C_{m}^{d}\frac{dV^{d}}{dt}=-g_{L}^{d}(V^{d}-E_{L}^{d})-g_{e}^{d}(t)(V^{d}-E_{e}^{d})-g_{i}^{d}(t)(V^{d}-E_{i}^{d})+\\ \;+I_{Ca}+I_{K_{Ca}}+w_{BAP}\sum_{k}\delta (t-(t_{k}+d_{BAP}))+\\ \;+I_{e}^{d}+g_{C}(V^{d}-V^{s})\\ \;\frac{d[Ca]}{dt}=\phi_{Ca}I_{Ca}+\frac{[Ca]-{\left[Ca\right]}_{0}}{\tau_{Ca}} \end{array}\right.\\ \left\{ \begin{array}{l} C_{m}^{s}\frac{dV^{s}}{dt}=-g_{L}^{s}(V^{s}-E_{L}^{s})+g_{L}^{s}{\text{Δ}}_{T}\exp\left(\frac{V^{s}-V_{th}^{s}}{{\text{Δ}}_{T}}\right)+\\ \;-g_{e}^{s}(t)(V^{s}-E_{e}^{s})-g_{i}^{s}(t)(V^{s}-E_{i}^{s})+\\ \;-g_{w}w+I_{e}^{s}-g_{C}(V^{s}-V^{d})\\ \;\tau_{w}\frac{dw}{dt}=a(V^{s}-E_{L}^{s})+b\sum_{k}\delta (t-t_{k})-w, \end{array}\right. \end{array}\right.$$

where *V*_*d*_ represents the membrane potential of the distal compartment, [*Ca*] the concentration of Ca2+ions in the distal compartment, *V*_*s*_ is the somatic membrane potential and *w* accounts for the Spike Frequency Adaptation. A somatic spike event is triggered when $V^{s}\geq V_{th}$, which defines the *t*_*k*_ spike time. *V*^*s*^ is set to the constant value *V*_*reset*_ during *t*_*k*_ < *t* ≤ *t*_*k*_ + *t*_*ref*_, while the distal compartment continues to integrate the dynamics defined by Equation 1 during this period. Figure 2 is the schematic representation of the Ca-AdEx neuron model in terms of an electronic circuit.

In principle, any kind of leaky integrate and fire with supporting spike frequency adaptation could be used for the somatic compartment. In our model, the soma follows the dynamics of an adaptive exponential integrate and fire neuron (AdEx, Gerstner et al., 2014) as described by the second set of equations in Equation 1. The parameters are described in detail in Supplementary Table S1.

The backpropagation-activated calcium spike (BAC firing, Larkum, 2013) is induced by the coincidental occurrence of a synaptic input to the apical dendrite and a *Na*^+^ spike triggered in the axon initial segment (AIS). This *Na*^+^ spike backpropagates via the soma to the Ca-HZ within the distal apical dendrite (BAP), where its depolarizing current adds to the local, subthreshold excitatory postsynaptic signal, thus triggering a dendritic Ca2+-spike in the Ca-HZ. Thus, the backpropagating *Na*^+^ spike effectively lowers the threshold for a local excitatory postsynaptic potential to trigger dendritic Ca2+-spike. The inward current of the dendritic Ca2+-spike is electrotonically conducted to the soma and AIS, where it causes a depolarizing wave that can trigger a high frequency burst of multiple action potentials, even if the apical excitatory postsynaptic potential (EPSP) alone would be subthreshold.

The activation of the calcium spike in the dendrite is the critical element for the BAC firing. To support this activation, we modeled a neuron implementing a voltage dependent Ca2+current and the [Ca2+] concentration dynamics within the apical dendritic hot-zone compartment. Additionally, a Ca2+-activated *K*^+^ current is included to re-polarize the dendritic membrane and thus terminate the Ca2+-spike.

The dendritic intracellular [Ca2+] dynamics has been modeled, as described in Gerstner et al. (2014), using the following equation:


(2) $$\begin{array}{l} \frac{d\left[Ca\right]}{dt}=\phi_{Ca}I_{Ca}+\frac{\left[Ca\right]-{\left[Ca\right]}_{0}}{\tau_{Ca}}, \end{array}$$

where [*Ca*]_0_ represents the baseline of the intracellular Ca2+concentration in mM, τ_*Ca*_ is the time constant of calcium extrusion in ms, *I*_*Ca*_ is the high voltage activated Ca2+current in the dendrite in pA and ϕ_*Ca*_ is a scaling factor.

Dendritic ionic currents are modeled using the Hodgkin-Huxley formalism. The high voltage activated Ca2+current (*I*_*Ca*_) has been modeled as in Larkum et al. (2004):


(3) $$\begin{array}{l} I_{Ca}=g_{Ca}mh\left(E_{Ca}-V\right), \end{array}$$

where *g*_*Ca*_ is the maximal calcium conductance in nS, *E*_*Ca*_ is the calcium reversal potential and *V* the membrane voltage, both in mV. The activation and inactivation variables, *m* and *h* respectively, are characterized by first-order kinetics:


(4) $$\begin{array}{l} \frac{dm}{dt}=\frac{m_{\infty }-m}{\tau_{m}}\text{and}\frac{dh}{dt}=\frac{h_{\infty }-h}{\tau_{h}}, \end{array}$$

where *m*_∞_ and *h*_∞_ are the corresponding steady state functions and τ_*m*_ and τ_*h*_ are their time constants in ms. The steady state functions are given by:


(5) $$\begin{array}{l} m_{\infty }=\frac{1}{1+exp\left(m_{slope}\left(V-\left(m_{half}\right)\right)\right)}\text{and}\\ h_{\infty }=\frac{1}{1+exp\left(h_{slope}\left(V-\left(h_{half}\right)\right)\right)}, \end{array}$$

with *m*_*slope*_ and *h*_*slope*_ representing the slope of the two functions and *m*_*half*_ and *h*_*half*_ representing the half activation/deactivation values in mV.

The Ca2+activated *K* current (*I*_*K*_*Ca*__) has been modeled as in Hay et al. (2011):


(6) $$\begin{array}{l} I_{K_{Ca}}=g_{K}m\left(E_{K}-V\right), \end{array}$$

where *g*_*K*_ is the maximal potassium conductance in nS, *E*_*K*_ is the potassium reversal potential and *V* is the membrane voltage, both in mV. *m* represents the activation variable described by the first order kinetics:


(7) $$\begin{array}{l} \frac{dm}{dt}=\frac{m_{\infty }-m}{\tau_{m}}. \end{array}$$

Here τ_*m*_ is the activation time constant of the Ca2+-activated *K*^+^ current in ms and *m*_∞_ is its activation steady state variable described by:


(8) $$\begin{array}{l} m_{\infty }=\frac{1}{1+{\left(\frac{Ca_{th}}{\left[Ca\right]}\right)}^{const_{K_{Ca}}}}, \end{array}$$

where *Ca*_*th*_ represents the Ca2+concentration threshold (in mM) for the Ca2+-activated *K*^+^ current and *const*_*K*_*Ca*__ is an exponential factor. The AdEx mechanism and the ionic currents are implemented within the NEST compartmental modeling framework (Section 2.4), allowing their incorporation in the somatic and Ca-HZ compartment, respectively.

In summary, a simple two-compartment Ca-AdEx neuron is described by Figure 2 and Equation 1. Supplementary Table S1 presents the complete set of parameters of the Ca-AdEx neuron identified in this work using the evolutionary approach described in the following sections.

### 2.2 Fitting the neuron model

An evolutionary algorithm has been used to fit the two-compartment Ca-AdEx neuron model within a multidimensional parameter space. More than 30 parameters have been fitted to ensure the desired behavior of the apical mechanism in the amplification regime. The search, optimized by the use of the L2L framework (see Section 2.2.2), is grounded in the definition of specific fitness functions that are used to constrain the evolution of the model during specific tasks (see Section 2.2.1).

Since the parameter set is identified using an evolutionary algorithm, we refer to it as the *genome* of the Ca-AdEx neuron model. At the initial time, a set of *individuals* (i.e., model configurations) is created, randomly extracting each parameter from a uniform distribution, carefully bounded to biologically plausible values. This set enters the evolutionary tool that, thanks to *mate, mutation*, and *genetic modification*, produces the next *generation* of individuals. The convergence of this mechanism is driven by the essential constraints of the fitness functions.

**Figure 3:**
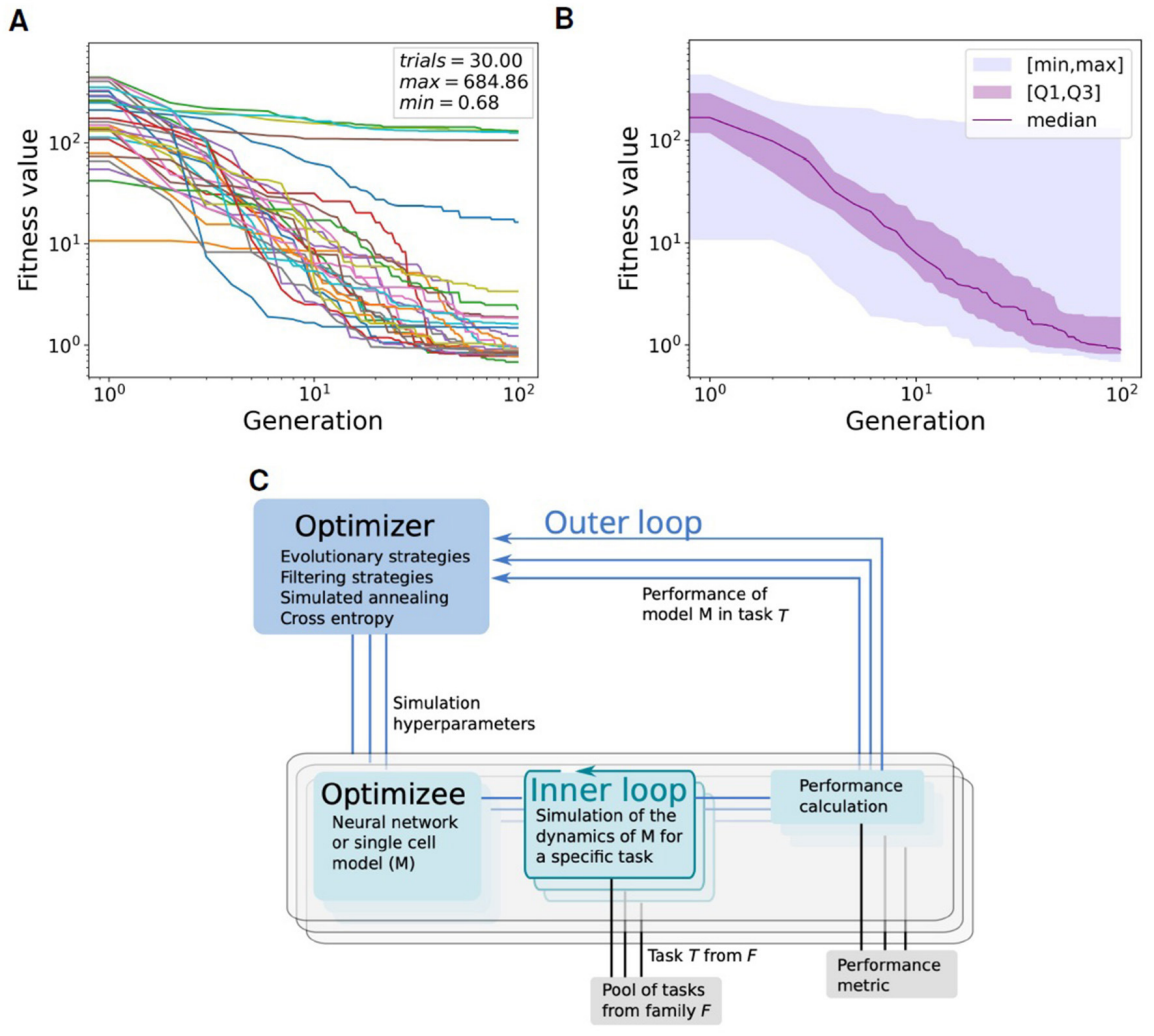
Convergence of the global fitness value within the Learning-to-learn (L2L) optimization framework. The global fitness, representing the sum of all single fitness functions return value, is plotted through all the 100 generations (including 100 individuals per generation) for 30 trials. **(A)** Plot of global fitness for each of the 30 single trials. **(B)** Median (purple), first and third quartile (purple shadow), within the [min-max] range (blue shadow) of the global fitness values over the 30 trials shown in **(A)**. **(C)** Two-loop scheme of Learning-to-learn (L2L): in the inner loop, a model is trained or simulated on a task from a family of tasks; a fitness function evaluates the performance of the model; the model parameters are optimized in the outer loop. Image provided by Yegenoglu et al. (2022).

Figures 3A, B illustrate the convergence of the evolution algorithm used for fitting the neural parameters. The convergence has been estimated over 30 trials, each one starting with a population of 100 random individuals and running for 100 generations.

#### 2.2.1 Fitness functions

Specific fitness functions have been devised to constrain the model. These functions aim to guide the evolutionary search within the parameter space toward optimal configurations, focusing on the identification of neurons that embody both the spiking frequency–stimuli relationships characteristic of apical mechanisms and a response to somatic-only stimulus that mirrors the behavior of a single-compartment AdEx neuron.

In our work, the model fitness is assessed considering two different optimization tasks based on the response of the Ca-AdEx neuron to different stimulation protocols: the response to *pulse stimuli* of a few milliseconds in duration and the response to *prolonged stimuli* lasting few seconds.

In the *pulse stimuli* task, the Ca-AdEx neuron model is designed to replicate the kind of experiment presented in Larkum et al. (1999), demonstrating the apical-amplification effects through the activation of BAC firing in response to a coincidence of basal and apical short-duration current injections. The goal is to emulate the experimental observations illustrated in Figure 1 of Larkum et al. (1999), that depicts the responses of a pyramidal neuron to four combinations of short duration current injections delivered to the distal apical dendrite and to the soma: 1- a subthreshold distal depolarizing current injection produces a minimal deflection of the somatic membrane voltage, without eliciting any spike; 2- a supra-threshold somatic current injection evokes a single action potential; 3- the combination of current injections as in 1- and 2-, separated by an interval of 5 ms, evokes a burst of action potentials; 4- to evoke a burst similar to the one produced in 3- using only dendritic input, a current larger than the one used in 1- and 3- is needed. Our *pulse stimuli* task is conceived to replicate these four cases.

Notably, the most interesting scenario, case 3-, involves injecting a brief (5 ms) rectangular current at just supra-threshold intensity into the somatic compartment, accompanied by a sub-threshold depolarizing current, shaped like an excitatory postsynaptic potential in the apical compartment (modeled with a beta-shaped current in our simulations) with a 5 ms delay (see Section 3.1). The threshold somatic current amplitude is calibrated to elicit a single spike in isolation, both in the experiments by Larkum et al. (1999) and in our simulations. Conversely, the injection of the subthreshold distal current alone does not produce any spike. The combination of these two currents activated the BAC firing mechanism, leading to a high-frequency burst of three spikes. To guide the model toward accurately responding to the four combinations of short-duration pulses, four fitness functions are employed. These functions aim to generate the correct number of spikes in short-duration bursts (see the *Pulse stimuli* section of Supplementary Table S3).

The second optimization task, the *prolonged stimuli* task, is motivated by the aim of supporting fast classification, recognition (and learning) of individual experiences. For example, considering images presented at video rate (i.e., more than 20 frames/s), neuron should react to individual perceptions lasting less than 50 ms. Firing at several tens of Hz is required to support classification/recognition capabilities at this rate. Learning can pose even stronger requirements. Indeed, a typical choice in spiking networks is to use Spike-Timing Dependent Plasticity (STDP). STDP models depend on the temporal relation between the spikes of presynaptic (*pre*) and postsynaptic (*post*) neurons (e.g., see Song et al., 2000; Gütig et al., 2003). A common choice, rooted on experimental evidence that started from the seminal works of Markram et al. (1997) and Bi and Poo (1998), is to model the amplitude of synaptic changes as proportional to *exp*(−|(*t*_*post*_−*t*_*pre*_|/τ_*STDP*_), where *t*_*post*_ and *t*_*pre*_ are the pre-synaptic and post-synaptic spike times. τ_*STDP*_ = 20 ms is the typical choice.

In the prolonged stimuli task the neuron model is subjected to pairs of long-lasting (2 s duration) DC current injections in the somatic basal compartment (*I*_*s*_) and in the dendritic apical compartment (*I*_*d*_). Combinations of somatic and distal dendritic current injections are kept constant for 2 s, followed by a 3 s period of zero input. The corresponding set of fitness functions is detailed in the *Prolonged stimuli* section of Supplementary Table S3. In this scenario, the computation of fitness functions relies on several different measures.

A first set of fitness functions is are set to ensure the correct behavior of the BAC firing. Initially, evaluations are made concerning the activation of Ca2+channels and their closure after the stimulus ends. Two additional fitness functions are used to discard neurons in which the calcium spike is activated with purely somatic currents and those showing calcium activation for very high currents.

An additional goal is to develop a two-compartment neuron that, when somatically stimulated, mimics an equivalent single-compartment AdEx. Therefore, we have introduced the *AdEx matching* fitness functions dedicated to this purpose. These functions are measured on a Ca-AdEx stimulated with increasing somatic currents *I*_*s*_ while keeping *I*_*d*_ = 0 and matched against the corresponding measures on single-compartment AdEx (see Supplementary Table S2 for the target AdEx parameters). The first function employs the Earth Mover's Distance (EMD) algorithm to compare the firing rate values of the two neurons. Additionally, we compare the first (rheobase) and the last firing rate values expressed by the single-compartment and two-compartment neurons within the observation range. Also, a measure of the coefficient of variation of inter spike intervals (ISIs) in response to somatic current is used to select neurons similar to the single-compartment AdEx neuron. Finally, a check on the neuron capacitance is performed, to drive the evolution toward a value as near as possible to the capacitance of the single-compartment AdEx model.

The set of fitness functions, *Gain & linearity of apical mechanism*, aims to ensure that the model exhibits a high, linear gain associated with the apical mechanism. Specifically, for the ν(*I*_*s*_, *I*_*d*_) transfer function, evaluations include: the firing rate following Ca2+opening for a distal-only stimulus (*I*_*s*_ = 0, *I*_*d*_), and the linearity in the increment of firing rate linked to calcium channel activation for increasing somatic and distal currents. Moreover, particular fitness functions focus on ensuring the monotonicity of the ν(*I*_*s*_, *I*_*d*_ = *const*) curves and the presence of the apical gain mechanism across the desired input domain: $I_{s}=0,..,I_{s}^{Max}$, $I_{d}=0,..,I_{d}^{Max}$.

A fitness function is dedicated to the *Exclusion of pathological configurations* for neurons exhibiting excessively high firing rates when stimulated within the predefined range of currents. Finally, an additional set of *Cautionary checks* is introduced to further constrain the neurons: the minimum value of the somatic membrane potential reached during stimulation, the value of the distal rheobase for *I*_*s*_ = 0, the occurrence of jumps in firing rate after calcium opening.

#### 2.2.2 The learning to learn framework

Learning-to-learn, or meta-learning (Thrun and Pratt, 2012, 1998), is an approach in machine learning aimed at enhancing learning performance through generalization. In a conventional learning setting, a program or algorithm is trained to perform a single task, evaluated by a specific performance metric. The algorithm performance improves as it is exposed to more training samples. After sufficient training, the algorithm or model can achieve high performance on new samples of the same task that it did not encounter during the training phase. This paradigm can be implemented as a two-loop structure, as shown in Figure 3C. In the inner loop, the program, also known as the *optimizee*, can adapt to learn a specific task from a family of tasks. These tasks may range from classification and inference to training multi-agents for complex problem-solving. A fitness function assesses the performance of the optimizee and yields a fitness value. This function is tailor-made for the task and must be precisely defined to effectively evaluate the optimizee. In the outer loop, the algorithm overall performance is enhanced by optimizing the hyper-parameters or parameters across a spectrum of tasks, facilitating the evolution of the entire system.

Yegenoglu et al. (2022) introduces an implementation of the learning-to-learn concept within a framework named *L2L*. In L2L, the outer loop is composed of various gradient-free optimization techniques based on meta-heuristics, including evolutionary algorithms or filtering strategies. In our case (see Supplementary Section 5), the GeneticAlgorithmOptimizer produces a set of optimizees for each generation using the strategies defined by the GeneticAlgorithmParameters function. All the optimizees are then evaluated on a task that includes the measure of fitnesses evaluated on a set of different stimulation scenarios, as described in Section 2.2.1.

The framework versatility allows for the execution of any algorithm or simulation, which can then be operated on anything from local machines to high-performance computing systems (HPCs). Thanks to the framework inherently parallel structure, multiple instances of the inner loop can be efficiently deployed on HPC systems. L2L necessitates only a performance measure and a set of parameters for optimization targets. It is developed in Python, is available as open-source, and adheres to an open development model.

For more information about the specific deployment used in this work please see the Supplementary material.

### 2.3 Modulating the apical-amplification, -isolation, and -drive regimes

A few parameters serve as simulation proxies for the effects of neuromodulation, facilitating transitions to apical-isolation-like and apical-drive-like regimes or modulating the apical-amplification behavior. Conceptual guidelines that have inspired the approach described here, summarizing experimental evidence, are Aru et al. (2020b) (about awake apical-amplification), Suzuki and Larkum (2020) (apical-isolation in anesthesia), and Aru et al. (2020a) (apical-drive and dreaming). As a proxy for neuromodulation (e.g., by cholinergic and noradrenergic neuromodulatory inputs), we propose to consider three contributions: 1- in the extreme apical-isolation case, we set to 0 the coupling conductance (*g*_*C*_) between the two compartments, to mimic the isolation of the distal apical dendrite demonstrated by Suzuki and Larkum (2020); 2- we change the Spike Frequency Adaptation coefficient *b* in Equation 1 (a classic modeling choice associated to cholinergic modulation, Destexhe, 2009); 3- we introduce a brain-state dependent shift of the leakage reversal potential, a crude approximation of: (3.1) the change of excitability of pyramidal layer V cells induced by noradrenaline on α1-noradrenergic receptors, that modulate potassium currents (Wang and McCormick, 1993) and, (3.2) the action of muscarinic ACh receptors that also change the amount of potassium current in layer V cortical neurons (Wang and McCormick, 1993).

**Figure 4:**
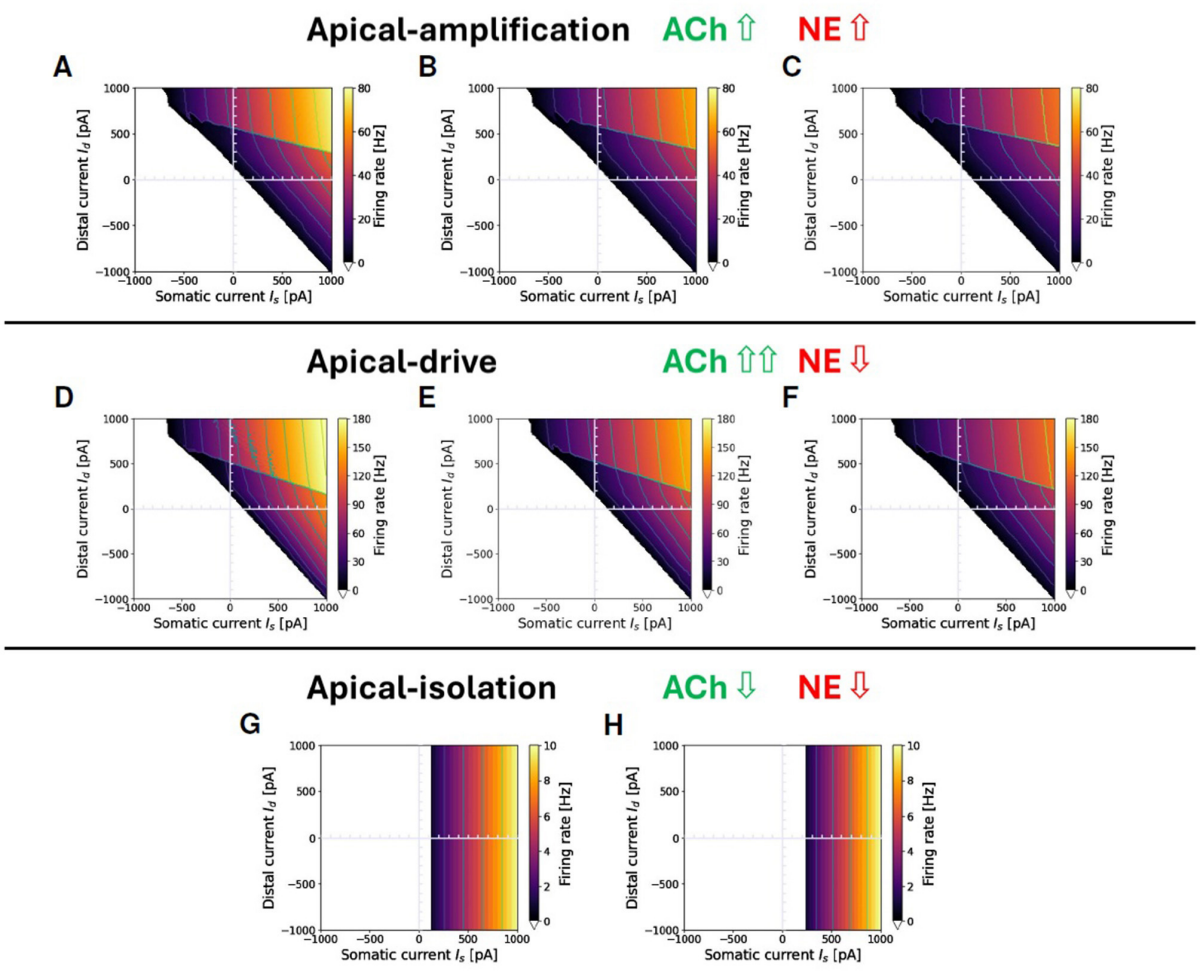
Proxies for ACh and NE modulation inducing a range of apical-amplification -isolation and -drive. Transfer functions showing different firing rates obtained with parameter tuning. Note that the values of maximum firing rates in color bars depend on the brain-state. Apical-amplification: starting from the Ca-AdEx parameters identified by the evolutionary search, the adaptation parameter has been set to: *b* = 40 **(A)**, *b* = 50 **(B)**, *b* = 60 **(C)**. Apical-drive: starting from the Ca-AdEx parameters, the somatic and distal reversal potentials are lowered of 2 mV and the adaptation parameter has been set to *b* = 10 **(D)**, *b* = 15 **(E)**, *b* = 20 **(F)**. **(G)** Apical-isolation: obtained using the Ca-AdEx parameters with *b* = 200, *g* = 0 and lowering somatic and distal reversal potentials of 5 mV. **(H)** Apical-isolation obtained using target AdEx parameters for the somatic compartment and Ca-AdEx parameters for the distal compartment, with *b* = 200, *g* = 0 and lowering somatic and distal reversal potentials of 5 mV.

Following these conceptual guidelines, we have performed an exploration in terms of neuromodulation in the three different brain states. The results are reported in Figure 4. Figures 4A–C represent a situation of apical-amplification. Starting from the neural parameters fitted using the L2L tool, we have investigated the effects of the changes in the adaptation parameter: *b* = 40 (Figure 4A), *b* = 50 (Figure 4B), and *b* = 60 (Figure 4C). While the behavior of the model remains substantially the same in the three cases, a global lowering of the firing rate is evident for growing values of the adaptation parameter, as well as a less evident firing rate jump in correspondence of the Ca2+activation. The apical-drive is obtained, as described above, by lowering of 2*mV* the somatic and distal reversal potentials and setting the adaptation parameter to *b* = 10 (Figure 4D), *b* = 15 (Figure 4E), and *b* = 20 (Figure 4F). Note that the firing rate in the apical-drive state is well higher than the one in the apical-amplification state.

Finally, Figures 4G, H describe two possible situations in apical-isolation. One of the goals of this work is the set-up of two-compartment neurons that, when stimulated only on the somatic compartment, behave like the AdEx single compartment reference neuron. This simplifies the replacement of single-compartment neurons in reference network simulations. Therefore, when considering the apical-isolation case, and the simulation of deep-sleep or anesthesia, a possible choice is to change all the parameters of the somatic compartment exactly at the value of the reference AdEx, because for *g*_*C*_ = 0 the spikes emitted by Equation 1 analytically reduce to those emitted by the soma for any distal stimulus, as prescribed by Suzuki and Larkum (2020) (see Figure 4H). Another plausible choice, when the primary interest is not a seamless replacement in existing single-spike simulations entering deep-sleep or anesthesia, is to maintain the somatic parameters at their nominal values (see Figure 4G).

### 2.4 Support for multi-compartment neurons in NEST

To leverage existing technology for the efficient simulation of recurrently connected spiking neural networks, we have integrated a general multi-compartment (MC) modeling framework into NEST. Generally, MC models can be represented as


(9) $$\begin{array}{l} C^{i}\frac{dV^{i}}{dt}=g_{L}^{i}\left(E_{L}^{i}-V^{i}\right)+\underset{c\in C^{i}}{\sum }I_{c}^{i}\left({\text{y}}_{c}^{i},V^{i}\right)+\underset{r\in R^{i}}{\sum }I_{r}^{i}\left({\text{y}}_{r}^{i},V^{i},S_{r}^{i}\right)\\ \;+\underset{j\in N^{i}}{\sum }g_{C}^{ij}\left(V^{j}-V^{i}\right), \end{array}$$

where *V*^*i*^ denotes the membrane potential in compartment *i*, *C*^*i*^ its capacitance, $g_{L}^{i}$ its leak conductance and $E_{L}^{i}$ the leak reversal potential. An arbitrary set $C^{i}$ of ionic channels may be present in compartment *i*. Their current $I_{c}^{i}\left({\text{y}}_{c}^{i},V^{i}\right)$ depends on the local membrane potential and a set of channel state variables ${\text{y}}_{c}^{i}$. Similarly, there may be an arbitrary set $R^{i}$ of synaptic receptors, whose current may depend on state variables ${\text{y}}_{r}^{i}$, the membrane potential and the presynaptic input spike train $S_{r}^{i}$. Finally, the compartment *i* is coupled to its neighbors $N^{i}$ through a coupling conductance $g_{C}^{ij}$. Due to the conservation of current, the coupling is symmetric, i.e., $g_{C}^{ij}=g_{C}^{ji}$. By identifying the compartments with the nodes of a graph and the neighbor couplings with the edges, the MC model is always a tree graph.

In simulation tools for detailed biophysical models, the continuous cable model of neuronal morphology is discretized spatially using the second order finite difference approximation (Carnevale and Hines, 2006), and the resulting system of equations takes the form of Equation 8. The number of compartments, or inversely their separation, is often chosen based on the electrotonic length constant. At a more abstract level, simplified multi-compartment (MC) models with two or three compartments are frequently utilized to represent elementary aspects of dendritic computation, with the parameters of Equation 8 being tuned by *ad-hoc* methods for the specific scientific problem under investigation (Pinsky and Rinzel, 1994; Clopath et al., 2007; Naud et al., 2014). Between these levels of detail, compartmental parameters can be derived from full morphologies through matrix algebra to simulate local computations (Wybo et al., 2021), or they can be explicitly tuned to replicate these computations (Pagkalos et al., 2023).

The compartmental model architecture in NEST accommodates all these use cases by offering API functionality that enables end users to directly set compartmental parameters and arrange them in a user-specified tree graph layout. Furthermore, it is designed to be straightforwardly extendable with ionic channels and receptor currents at the C++ level.

The system is discretized in time using the Crank-Nicolson scheme:


(10) $$\begin{array}{l} C^{i}\frac{V^{i}\left(t+h\right)-V^{i}\left(t\right)}{h}=\frac{F^{i}\left(V^{i}\left(t\right)\right)+F^{i}\left(V^{i}\left(t+h\right)\right)}{2}, \end{array}$$

where *F*^*i*^ represents right-hand side of Equation 8. It is important to note that this method is implicit in the voltage: *F*^*i*^(*V*^*i*^(*t* + *h*)) needs to be Taylor expanded so that all terms containing *V*^*i*^(*t* + *h*) (∀*i* ∈ MC) can be moved to the left-hand side. The resulting matrix equation is then solved efficiently through the Hines algorithm (Hines, 1984). For the state variables of ionic channels and receptor currents, we use the widely used leap-frog scheme: a state variable *y* is computed at $t+\frac{h}{2}$, and thus has this value in both *F*^*i*^(*V*^*i*^(*t*)) and *F*^*i*^(*V*^*i*^(*t*+*h*)). Conversely, to compute the time evolution of a state variables from $t+\frac{h}{2}$ to $t+\frac{3h}{2}$, the voltage *V*^*i*^(*t*+*h*) is taken to be constant over this time-step.

If the state variable follows the general Hodgkin-Huxley formalism, i.e.,


(11) $$\begin{array}{l} \frac{dy}{dt}=\frac{y_{\infty }\left(V\right)-y}{\tau_{y}\left(V\right)}, \end{array}$$

the value at time $t+\frac{3h}{2}$ follows from integrating this equation as an initial value problem starting from $y\left(t+\frac{h}{2}\right)$, which has the analytical solution:


(12) $$\begin{array}{l} y\left(t+\frac{3h}{2}\right)=Py\left(t+\frac{h}{2}\right)+\left(1-P\right)y_{\infty }\left(V\left(t+h\right)\right),\\ \text{with}\\ P=\exp\left(-\frac{h}{\tau_{y}\left(V\left(t+h\right)\right)}\right). \end{array}$$

For state variables that do not depend on the voltage, as is often the case for those governing the synaptic conductance after spike arrival, efficiency is enhanced by pre-computing the propagator *P*.

### 2.5 Semi-simplified morphological neuron model with NMDA spikes

To demonstrate the potential of the MC modeling framework, we have integrated Ca-AdEx into a neuron model that also includes dendritic compartments with NMDA-driven non-linearities, based on an L5PC morphology. This morphology is taken from Hay et al. (2011) and implemented in NEAT (Wybo et al., 2021). We have opted for a passive dendritic membrane, i.e., without any voltage-gated ionic channels (i.e., no voltage-gated *Na*^+^, *K*^+^, Ca2+, or HCN channels), as those channels only weakly influence NMDA-spike generation (Major et al., 2008). Also, somatic channels have not been added, as spike generation is implemented by the AdEx mechanism anyway. The physiological parameters recommended by Major et al. (2008) are adopted to replicate the amplitudes of glutamate-uncaging evoked NMDA-spikes in L5PC dendrites and somata, combined with a spine correction as in Rhodes (2006). Specifically, we use a membrane capacitance of 0.8 μF/cm^2^, which is increased by a factor 1.92 to account for spine surface in dendrites with a radius smaller than 0.6 μm. The axial resistance is set at 100 Ω × cm for smooth dendrites, and 120 Ω × cm for spiny dendrites, while the specific membrane conductance is 100 μS/cm^2^, and the leak reversal potential is fixed at −75 mV.

This model is then further simplified into a version with 6 distal apical and 8 distal basal compartments, using a previously developed systematic morphological simplification methodology (Wybo et al., 2021). These compartments receive glutamatergic (AMPA+NMDA) as well as GABAergic synapses, in addition to the soma and a Ca-HZ compartment located where the main apical trunk splits into an apical tuft of multiple branches. For technical reasons, all bifurcation sites in between any of those compartments are added automatically by the simplification procedure (see Section 3.5, Wybo et al., 2021). The parameters of the reduced model that also featured in the Ca-AdEx optimization procedure (such as the leak and capacitance of the soma and Ca-HZ compartments, as well as their coupling) are overwritten by those obtained through the optimization, and the other optimized parameters of the Ca- and AdEx-mechanisms are added as well.

The resulting model is then stimulated with input current steps, the BAC-firing protocol and Poisson distributed synaptic inputs (see Section 3.5). For the BAC-firing protocol, we use a somatic current step amplitude of 750 pA and a double exponential input current at the Ca-HZ compartment with τ_*r*_ = 1 ms and τ_*d*_ = 10 ms, and a maximal amplitude of 1500 pA. For the Poisson synaptic inputs, AMPA and GABA receptors are simulated as the product of a double exponential conductance window (Rotter and Diesmann, 1999) *g* and a driving force:


(13) $$\begin{array}{l} i_{\text{syn}}=g\left(e_{r}-v\right),\text{ with }g=wn\left(\tau_{r},\tau_{d}\right)\left(e^{-t/\tau_{d}}-e^{-t/\tau_{r}}\right). \end{array}$$

Here, *e*_*r*_ is the synaptic reversal potential, τ_*r*_ and τ_*d*_ are the synaptic rise and decay time constants, and *n* a normalization constant that depends on τ_*r*_ and τ_*d*_ and normalizes conductance window *g*, so that its peak value is equal to the synaptic weight *w*. AMPA rise and decay times are τ_*r*_ = 0.2ms, τ_*d*_ = 3ms and AMPA reversal potential is *e*_*r*_ = 0mV, whereas for GABA, we have τ_*r*_ = 0.2ms, τ_*d*_ = 10ms and *e*_*r*_ = −80mV. NMDA currents (Jahr and Stevens, 1990) are implemented as:


(14) $$\begin{array}{l} i_{\text{syn}}=g\sigma \left(v\right)\left(e_{r}-v\right), \end{array}$$

with τ_*r*_ = 0.2ms, τ_*d*_ = 43ms, and *e*_*r*_ = 0mV, while σ(*v*)—the channel magnesium block—has the form (Behabadi and Mel, 2014):


(15) $$\begin{array}{l} \sigma \left(v\right)=\frac{1}{1+0.3e^{-0.1v}}. \end{array}$$

The synaptic weight (i.e., maximum value of the conductance window) for the AMPA component of AMPA + NMDA synapses is set at 1 nS, and the maximal value of the NMDA window is twice that of the AMPA window (NMDA ratio of 2). GABA synapses also have a weight of 1 nS. While for the AMPA+NMDA synapses a multitude of Poisson input rates are probed as part of the scan, the Poisson input rate to the GABA synapses is fixed at 20 Hz.

### 2.6 Fitting the transfer function

**Figure 5:**
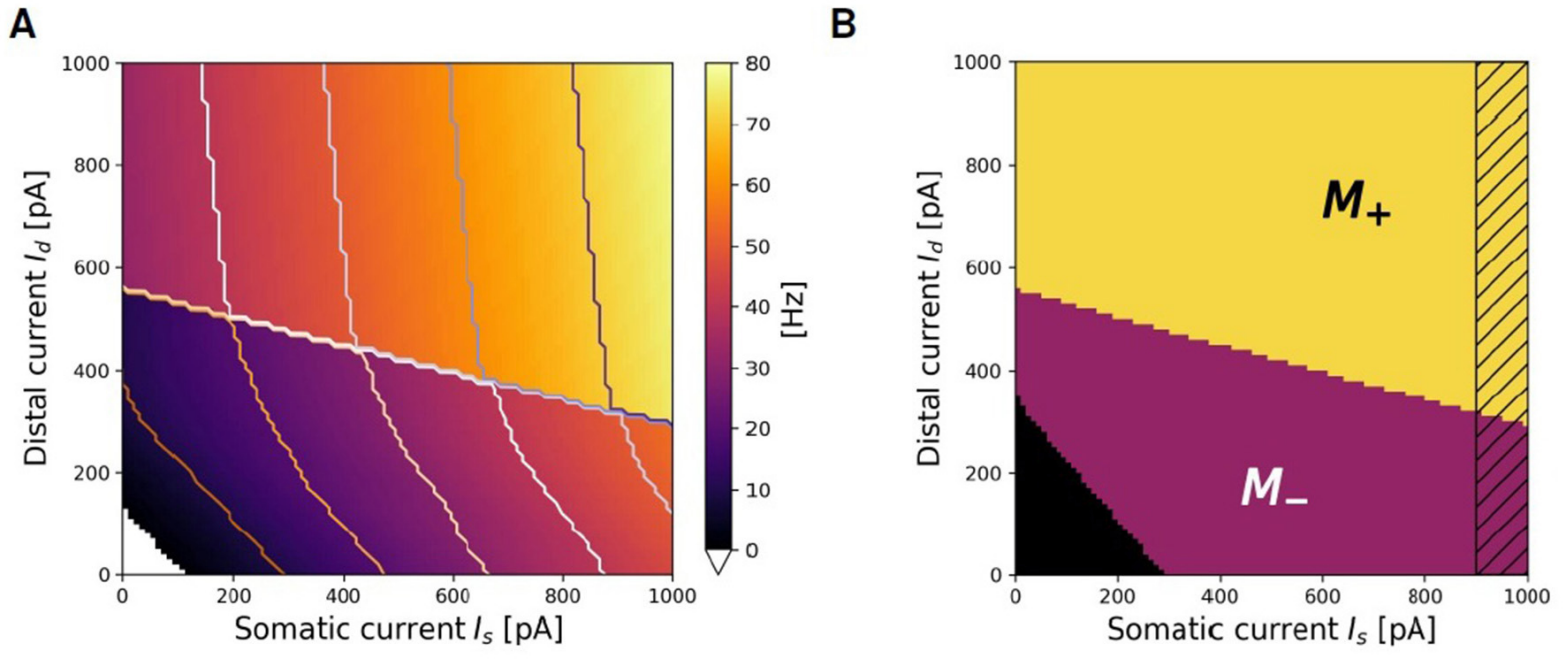
Search for approximating planes. **(A)** Representation of the firing rate ν(*I*_*s*_, *I*_*d*_) of the Ca-AdEx spiking neuron in response to combinations of somatic (*I*_*s*_) and distal (*I*_*d*_) currents. **(B)** Algorithmic identification of *M*_+_ and *M*_−_ regions from spiking simulation results.

Figure 5A illustrates ν(*I*_*s*_, *I*_*d*_), the firing rate of the exemplary two-compartment Ca-AdEx neuron identified by the evolutionary search algorithm in response to various combinations of constant somatic and distal currents. The regularity observed in the contour lines of equal firing rate suggests the potential for simplified approximate representations of the transfer function. This section outlines the method employed to derive such an approximation. Two distinct regions of low and high firing rate are discernible in Figure 5A, seemingly demarcated by a straight line. Hereafter, we use the index *i* ∈ {−, +} to denote the regions of lower or higher firing rates, respectively. In the + region, contour levels of equal firing rate appear to be linear, parallel, and evenly spaced, indicating that the transfer function could be approximated by a plane. For each (*I*_*s*_,*I*_*d*_) pair, the simulation identifies the activation of the High Voltage dependent Ca2+channel, resulting in a Boolean mask *M*_+_(*I*_*s*_, *I*_*d*_) that delineates the activation region associated with high firing rates (refer to Figure 5B).

Fitting planes ν_+_ are defined by


(16) $$\begin{array}{l} \nu_{+}\left(I_{s},I_{d}\right)=a_{+}I_{s}+b_{+}I_{d}+d_{+}, \end{array}$$

and their parameters (*a*_+_, *b*_+_, *d*_+_) have been identified in this work using the *LinearRegression* class from the *sklearn.linear_model* Python module (release 1.0.2). The same procedure returns the plane fitting the region of low activity *M*_−_ (i.e., the lower part of Figure 5A), where the contour lines are also approximately linear and evenly spaced for firing rates above a threshold ν_*low*_.

The selection of an appropriate ν_*low*_ frequency is motivated by the aim of modeling the learning advantages associated with apical-amplification mechanisms, particularly in scenarios where external stimuli change at a fast rate. For instance, detailed memorization of individual images at video rate (i.e., more than 20 frames/s), should happen for exposure to individual perceptions lasting less than 50 ms. τ_*STDP*_ = 20 ms is the typical choice. In a time interval of 50 ms, neurons firing at ν_*low*_ = 10 Hz would induce in the connecting synapses a single, small amplitude synaptic modification, due to the time dependent exponential decay of STDP rules. Apical amplification supports significantly higher firing rates. Therefore, capturing the regime of lower firing rates with extreme precision is not critical.

The *M*_−_(*I*_*s*_, *I*_*d*_) Boolean mask is defined by the points where the simulation indicates that *M*_+_(*I*_*s*_, *I*_*d*_) = =*false ANDν*(*I*_*s*_, *I*_*d*_)>ν_*low*_. The search for fitting planes can be done in the *M*_−_ region, employing the same algorithm used for *M*_+_, producing a ν_−_(*I*_*s*_, *I*_*d*_) approximating plane. To mitigate potential non-linearity at the boundaries of the region of interest, the Boolean masks excludes the region *I*_*s*_>*I*_*th*_, (illustrated as a dashed band in Figure 5B). Errors (in *Hz*) between the planar fit and the simulated transfer function are reported in Supplementary material.

The construction of ν_*F*_(*I*_*s*_, *I*_*d*_), the simplified description over the entire range of *I*_*s*_, *I*_*d*_ currents of the ν produced by spiking simulations, requires also a proper definition of the curve $I_{d}^{H}\left(I_{s}\right)$ that separates the *M*_+_ from the *M*_−_ regions. $I_{d}^{H}\left(I_{s}\right)$ is the amount of distal current required to trigger a high firing regime (*H*) given a fixed value of somatic *I*_*s*_ current. The linear fit of the data representing the boundary between *M*_+_ and *M*_−_ leads to the definition of the parameters $\theta_{m}^{H}$, the slope of the fitting line, and $\theta_{q}^{H}$, its offset. The resulting approximating line is expressed as:


(17) $$\begin{array}{l} I_{d,F}^{H}\left(I_{s}\right)=\theta_{m}^{H}I_{s}+\theta_{q}^{H}. \end{array}$$

The rheobase of the fitting function is defined by the combinations of currents that satisfy the condition ν_−_(*I*_*s*_, *I*_*d*_) = 0, this results in the line:


(18) $$\begin{array}{l} I_{d,F}^{\rho }\left(I_{s}\right)=\theta_{m}^{\rho }I_{s}+\theta_{q}^{\rho }. \end{array}$$

In summary, three planes (ν_0_ = 0, ν_−_(*I*_*s*_, *I*_*d*_) and ν_+_(*I*_*s*_, *I*_*d*_)) are identified by the algorithm to approximate the activity in each region. The active approximated domain is limited/bounded by:


(19) $$\begin{array}{l} {\text{Θ}}_{H}\left(I_{s},I_{d}\right)=\text{Θ}\left(I_{d}-I_{d,F}^{H}\left(I_{s}\right)\right). \end{array}$$

The passive approximated domain is given by the product of two Θs, namely:


(20) $$\begin{array}{l} {\text{Θ}}_{\rho }\left(I_{s},I_{d}\right)=\text{Θ}\left(I_{d}-I_{d,F}^{\rho }\left(I_{s}\right)\right) \end{array}$$

and


(21) $$\begin{array}{l} \text{Θ}\left(-I_{d}+I_{d,F}\left(I_{s}\right)\right)=\left(1-{\text{Θ}}_{H}\left(I_{s},I_{d}\right)\right). \end{array}$$

Finally, the fitting function that spans the entire domain, as determined by the algorithm, is:


(22) $$\begin{array}{l} \nu_{F}\left(I_{s},I_{d};\nu \right)={\text{Θ}}_{\rho }\left(1-{\text{Θ}}_{H}\right)\cdot \nu_{-}+{\text{Θ}}_{H}\cdot \nu_{+}. \end{array}$$

This is referred to as *ThetaPlanes* in the following.

## 3 Results

### 3.1 Response to pulse stimuli

**Figure 6:**
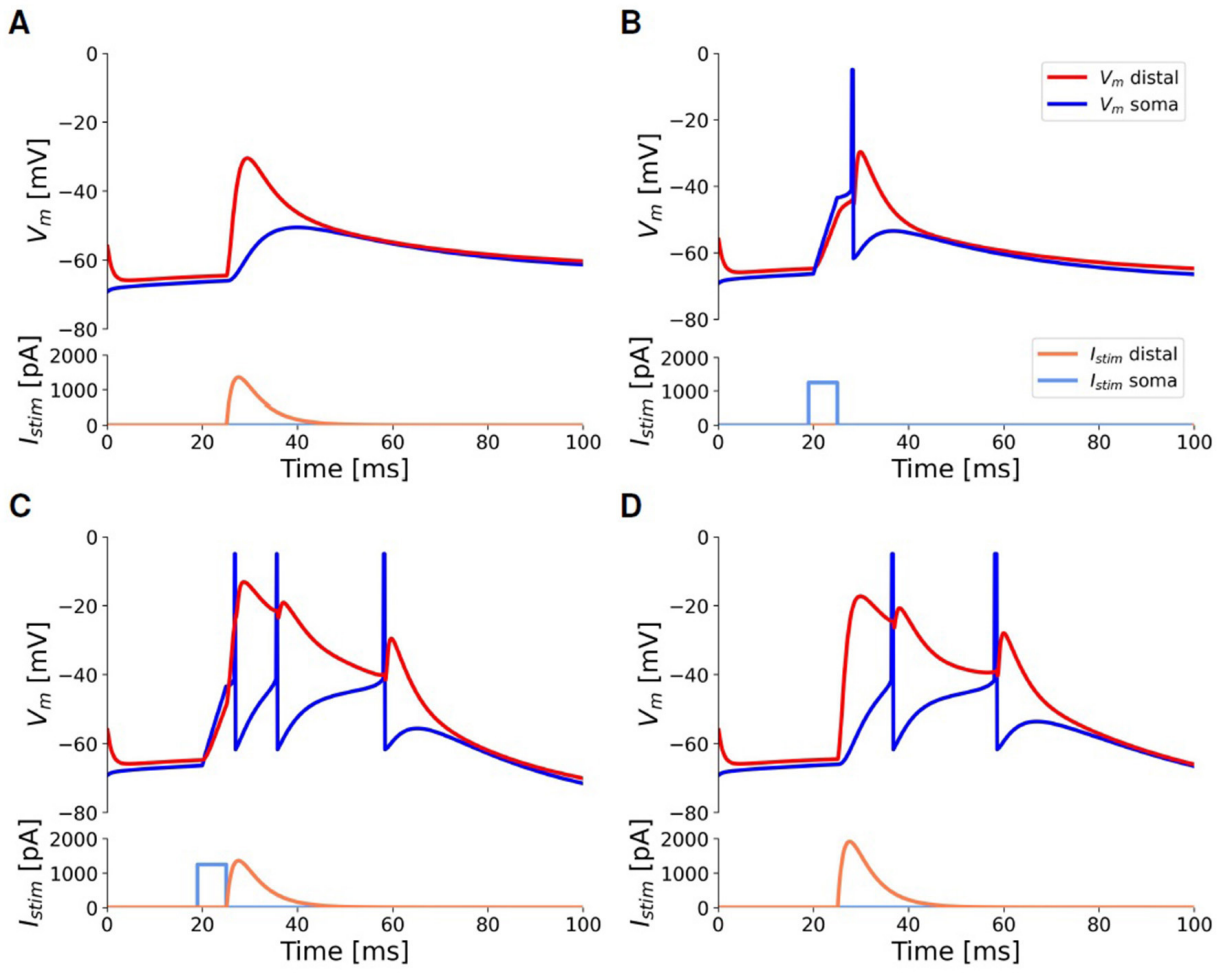
Responses of the selected neuron model to injected input currents of short duration, according to the *pulse stimuli* task outlined in Section 2.2.1, to simulate the experiments by Larkum et al. (1999). All panels share scale bars and legends. Blue: somatic membrane voltage. Red: apical membrane voltage. Light-blue: somatic step current injection. Orange: beta-shaped distal apical current injection. **(A)** A beta-shaped current injection of 1,345 pA (peak amplitude) at the apical compartment produces a deflection of only 15 mV at the soma without eliciting any spike (AP). **(B)** A threshold somatic current injection (1,150 pA, duration 5*ms*) evokes only a single AP. **(C)** The combination of the threshold somatic current as in **(B)** followed by the apical current as in **(A)** activates the BAC firing mechanism and evokes a burst of three APs. **(D)** To obtain a burst using only distal injection, a current of at least 1, 830 pA is required.

Figure 6 illustrates the behavior of the fitted model in response to depolarizing current injections of short duration (a few milliseconds), according to the protocol for the *pulse stimuli* task outlined in Section 2.2.1, which replicates the one used in the experiments by Larkum et al. (1999). A subthreshold distal depolarizing current injection, modeled as a beta function to mimic an excitatory postsynaptic potential (EPSP), slightly deflects the somatic membrane potential but does not trigger any spikes (Figure 6A). A just supra-threshold somatic current injection evokes a single action potential (AP) that “back-propagates” through the apical dendrite: this results in a depolarization of the apical dendrite but is not sufficient to initiate a calcium spike (Figure 6B). The concurrent application of the previously described somatic and dendritic current injections triggers a burst of two or three spikes at the soma: the single action potential, induced by the somatic input, back-propagates into the apical dendrite, thus depolarizing the Ca-HZ. When coupled with the subthreshold distal input, this facilitates the initiation of the dendritic calcium spike (Figure 6C). To generate a similar burst with only a distal input, a higher peak current value must be supplied, as demonstrated in the example of Figure 6D. When combining somatic and dendritic currents as in Figure 6C, the dendritic current is introduced with a delay of 5 ms relative to the somatic one. Analyzing how the response of the neuron depends on this delay is not covered in this work, but it will be considered for further optimization of the neuron.

### 3.2 Response to prolonged stimuli: compact geometric description of the transfer function

**Figure 7:**
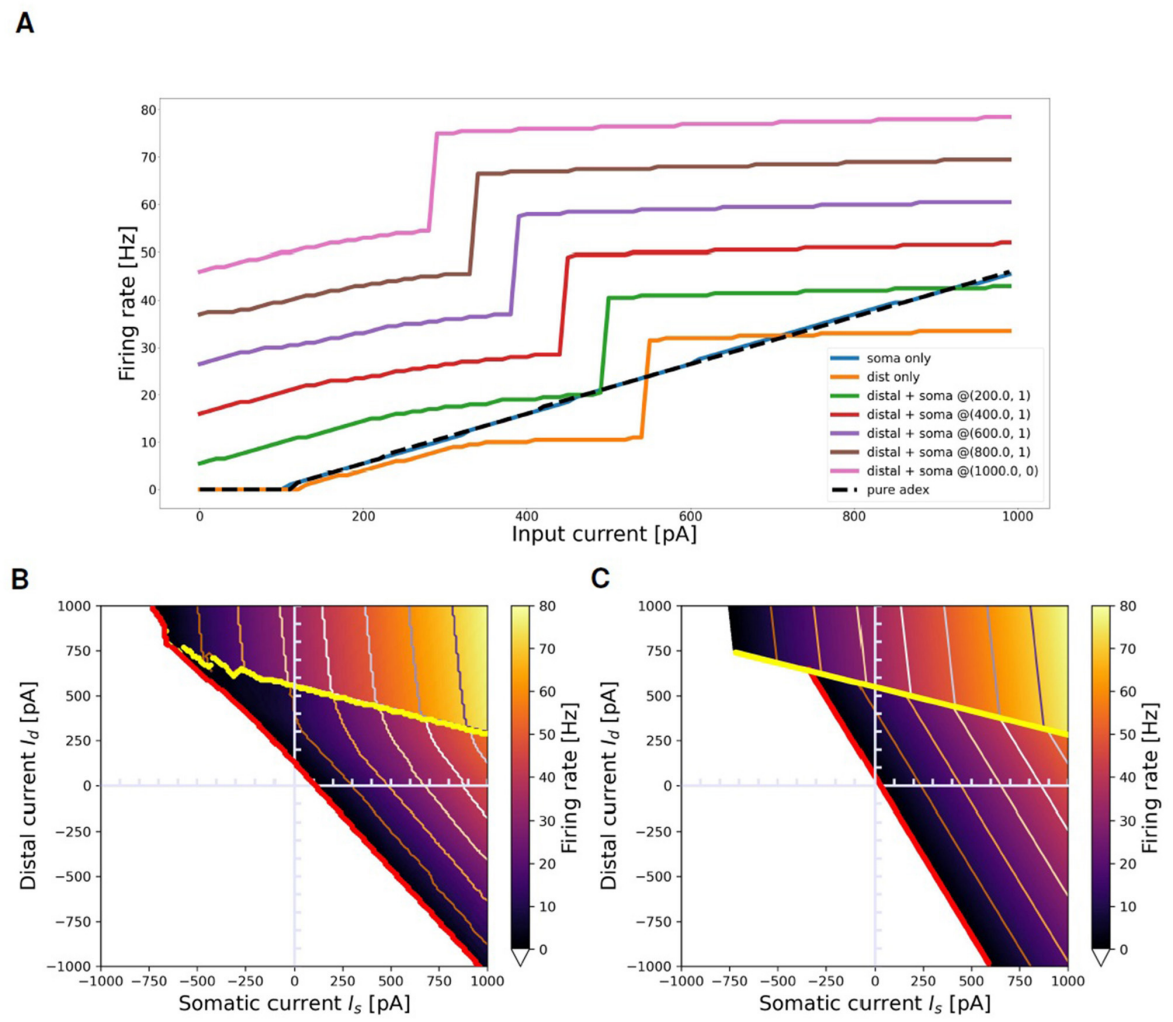
Transfer function of selected neuron and its approximation with ThetaPlanes. **(A)** Current to rate response to DC inputs delivered to different compartments: pure somatic current (blue), pure distal current (orange), combination of somatic and distal current (other colored lines) plotted for increasing distal currents at fixed somatic current. The dashed black line represents the transfer function of the AdEx neuron used as the target reference for the fitness function. **(B)** Transfer function of the neuron in the 2-D plane defined by somatic and distal input DC currents; in red the rheobase and in yellow the transition line between passive and active calcium regimes, respectively expressing a lower and an higher firing rate. **(C)** ThetaPlanes approximating the transfer function.

Figure 7 summarizes the main characteristics of the selected neuron in response to the *prolonged stimuli* task, outlined in Section 2.2.1. Figure 7A illustrates the neuron dynamics for specific distal and somatic input currents. The orange line depicts the firing rate when the neuron is stimulated solely with a distal current. The activation of the BAC firing mechanism is indicated by the sharp increase in the firing rate observed at 550 pA in the orange line: beyond this threshold, even without somatic input, a dendritic calcium spike is initiated, and the neuron enters into the active regime, wherein the mechanism of apical-amplification becomes apparent. The other curves illustrate the neuron responses when stimulated with a combination of currents injected into both the soma and the apical dendritic compartment, plotted for increasing dendritic current injections (*I*_*d*_) at fixed somatic current injections (*I*_*s*_). The visible jump in these curves corresponds to the neuron entering the active regime, a state reached when the combined effect of the two input currents is sufficient to trigger the dendritic calcium spike. As the value of the constant somatic current increases, the transition to the active regime occurs at progressively lower dendritic input currents. The blue line represents the scenario where *I*_*d*_ = 0: neither the calcium spike nor the BAC firing mechanism is triggered, and within the analyzed range, the neuron behaves similarly to the pure AdEx model against which the two-compartment model has been fitted (indicated by the black dashed line).

Figure 7B displays the firing rate of the neuron when stimulated with combinations of somatic and distal currents (ν(*I*_*s*_, *I*_*d*_)). Three distinct regions are identifiable: the area below the red line, where the firing rate equals 0 for every input current combination; an area of low firing rates situated between the red and yellow lines; and an area of high firing rates above the yellow line, indicating the triggering of the calcium spike and the activation of the apical-amplification mechanism (active regime). The red line denotes the neuron rheobase, while the yellow line signifies the transition from the passive to the active regime.

As discussed in the Section 2, by examining the firing rate ν of the multi-compartment neuron in the plane of somatic (*I*_*s*_) and distal (*I*_*d*_) input currents, we can create a simplified model at a significantly higher level of abstraction. This is achieved through the definition of two fitting planes, one for the apical-amplification zone and another for the lower activity region of the neuron transfer function. Therefore, ν can be piece-wise by planes separated by lines, resulting in the *ThetaPlanes* transfer function:


(23) $$\begin{array}{l} ThetaPlanes\left(I_{s},I_{d};\nu \right)={\text{Θ}}_{\rho }\left(1-{\text{Θ}}_{H}\right)\cdot \nu_{-}+{\text{Θ}}_{H}\cdot \nu_{+}. \end{array}$$

Figure 7C displays such approximating function, while Supplementary Table S4 lists the parameters that define the *ThetaPlanes* function and their values for the fitted configuration: the *v*_−_(*I*_*s*_, *I*_*d*_) and *v*_+_(*I*_*s*_, *I*_*d*_) planes, the transition line to high firing rates and the rheobase.

### 3.3 Wakefulness, NREM and REM specific apical mechanisms

**Figure 8:**
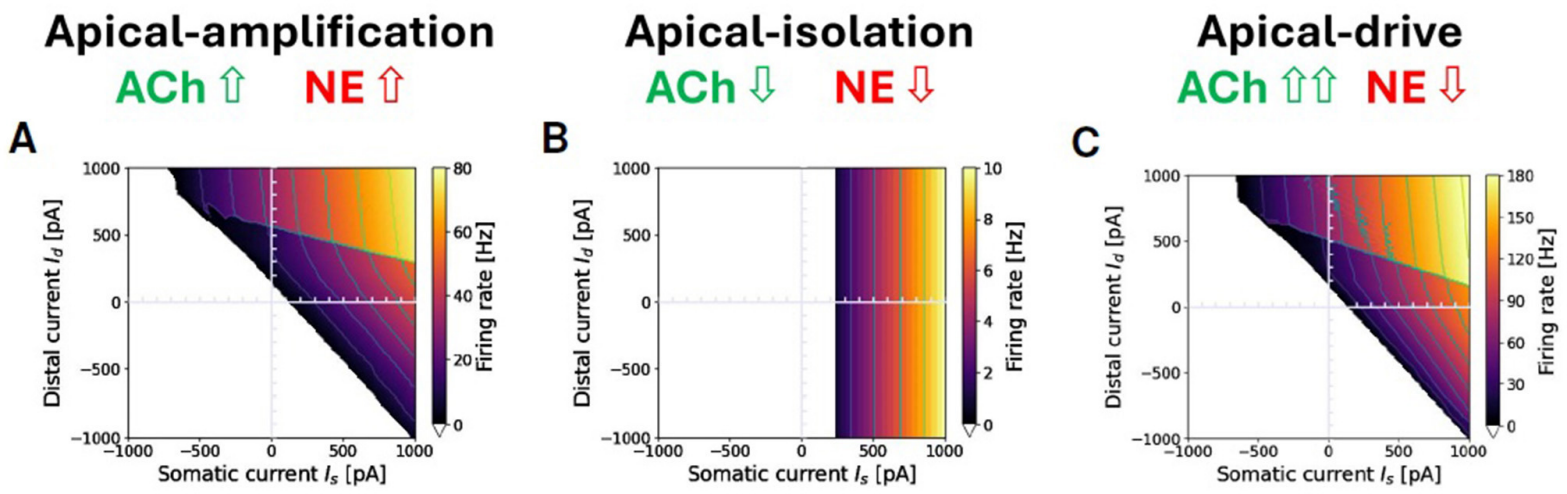
Apical-amplification, -isolation, and -drive: exemplary ν(*I*_*s*_, *I*_*d*_) firing rates in the three regimes. **(A)** Apical-amplification (wakefulness): neuron based on Ca-AdEx parameters identified by the evolutionary search. **(B)** Apical-isolation (NREM deep-sleep): obtained using target AdEx parameters for the somatic compartment and Ca-AdEx parameters for the distal compartment, with the following changes: *b* = 200, *g*_*C*_ = 0, somatic and distal reversal potentials decreased of 5 mV. **(C)** Apical-drive (REM sleep): neuron with Ca-AdEx parameters with the following changes: *b* = 10, somatic and distal reversal potentials decreased of 2 mV. Note: max ν is very different in the tree regimes: well over 100 Hz in apical-drive, up to 80 Hz in -amplification and about 10 Hz in -isolation. Also, the jump between the high-firing rate *M*_+_ and the *M*_−_ regions spans from tens of Hz in the apical-drive regime down to a few Hz in the -isolation regime.

Figure 8 illustrates three modulation of the simulation proxies of colinergic (ACh) and noradrenergic (NE) actions that alter the transfer function of the exemplary two-compartment neuron discussed throughout this paper in different brain-states. Specifically, Figure 8A depicts a representative awake apical-amplification configuration; Figure 8B presents a configuration tailored to simulate the NREM sleep apical-isolation regime, and Figure 8C showcases a setting related to the apical-drive configuration, which is expected to be associated with a REM sleep regime. See Section 2.3 for details and refences.

### 3.4 Computational cost of the Ca-AdEx model

The comparison between the computational cost of the single compartment AdEx neuron and that of the two compartment Ca-AdEx neuron is measured by simulating two balanced Brunel-like networks (Brunel, 2000). Both networks include 10000 excitatory neurons and 2,500 inhibitory neurons, in proportion 4:1, with an inh/exc synaptic weight ratio *g* = 5 and 1,250 input synapses per neuron. The AdEx and Ca-AdEx networks are both set in an asynchronous regime at about 1.23 Hz (see Table 1). Wall-clock execution times are measured for a biological simulation time of 10 seconds of asynchronous activity. Then they are averaged over eight trials per neuron model (different seeds for both network building and noise generation). For each trial (within each configuration), the execution time is normalized to the mean network activity rate. This results in a comparable time in terms of execution speed moving from single-compartment to two-compartment model. This is not surprising, given that the cost of a neural network simulation is usually dominated by spike exchange and simulation of synaptic activity -in this case 1,250 synapses per neuron- and the addition of a single distal compartment implies the addition of a few arithmetic operations per time-step. Simulations have been executed using the NEST 3.3 engine, parallelized on 32 threads and run on a dual-socket server equipped with an AMD EPYC 7302 16-Core Processor per socket, clocked at 3 GHz.

**Table 1:** Comparison of the computational cost: single-compartment AdEx vs. two-compartment Ca-AdEx.

**Metrics**	**AdEx**	**Ca-AdEx**
Mean network rate [Hz]	1.23 ± 0.03	1.23 ± 0.03
<Exec time/mean rate> [s/Hz]	57.42 ± 0.56	59.60 ± 1.12

Balanced Brunel-like network including 12,500 neurons with 1,250 incoming synapses per neuron, simulated for 10 seconds of biological asynchronous activity.

### 3.5 Extending the two-compartment layout

**Figure 9:**
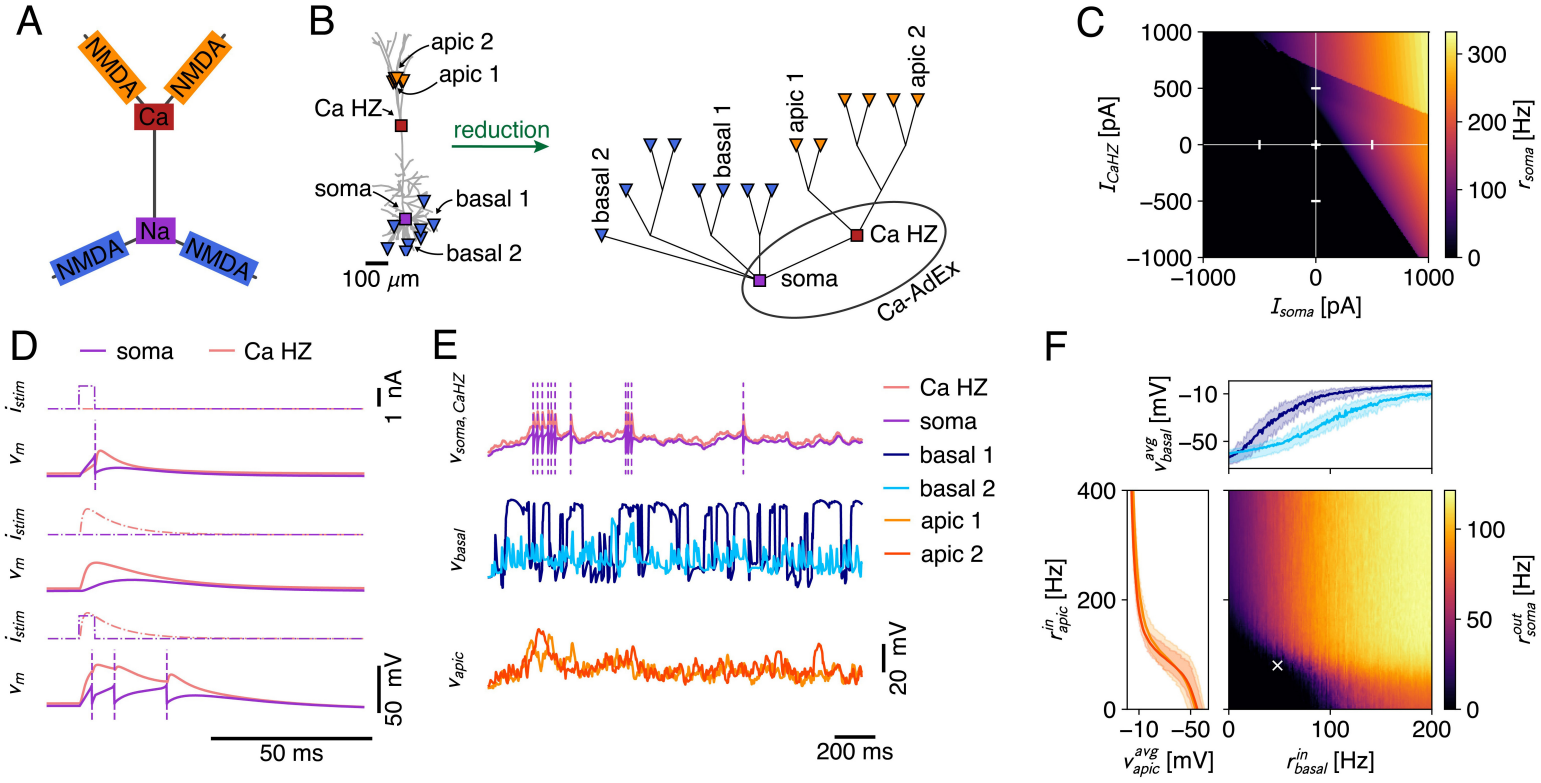
**(A)** Canonical view of the interplay between dendritic non-linearities (Larkum et al., 2009). NMDA spikes in distal apical branches (orange) elicit Ca-spikes (red) that result in somatic burst firing (purple), whereas basal NMDA-spikes (blue) directly influence somatic output generation. **(B)** Schematic of the creation process of the multicomp AdEx model. A passive morphology (left) with 16 locations (8 basal sites at ~200 μm from the soma [blue triangles], 6 apical tuft sites at ~1, 000 μm [orange triangles], the Ca-HZ where the apical trunk bifurcates [red square], and the soma [purple square]) is reduced to a simplified compartmental model (right) using the NEAT toolbox (Wybo et al., 2021). The Ca-hotzone and soma are then equipped with the Ca-spike generation mechanism and the AdEx mechanisms, respectively, where parameters are identical to the two-compartment model. Labeled apical and basal sites are those for which traces and mean activations are shown in **(E, F)**. **(C)** Firing rate response to input current steps applied to the soma and the Ca-hotzone compartment (same stimulation paradigm as in Figure 7B). **(D)** Simulation of the BAC-firing protocol, where a single output is generated in response to a somatic input pulse (top), no output is generated in response to a Ca-hotzone input (middle), and three output spikes are generated in response to the pairing of inputs (bottom). **(E)** Exemplary traces for stimulation of the model with Poisson inputs that impinged on AMPA+NMDA synapses located at the basal and apical sites. Purple dashed lines indicate spike times. **(F)** Firing rate response to increasing input rates to the apical and basal dendritic sites. Axes show the input rate to the individual dendritic sites (input rates equal across apical resp. basal sites). Inset plots show the average membrane potential in two exemplar apical (left) and basal (top) compartments [same sites as in **(B, E)**]. The min-max envelope shows the range of values obtained over all activation levels of the other area (i.e., apical vs. basal). The white cross marks the input rates shown in **(E)**.

While the Ca^2+^-spike is a well-known mechanism for coupling distal apical and peri-somatic inputs, local integration in thin dendritic branches is shaped by the NMDA-receptor (Figure 9A). Through the voltage dependent unblocking of the NMDA-receptor channel (MacDonald and Wojtowicz, 1982; Jahr and Stevens, 1990), coincident inputs to dendritic branches supra-linearly add, and the resulting events are known as NMDA-spikes (Schiller et al., 2000; Major et al., 2008, 2013). These NMDA-spikes are the primary biophysical mechanism that underlies the compartmentalization of the dendritic tree into semi-independent computational subunits (Behabadi and Mel, 2014; Wybo et al., 2019; Beniaguev et al., 2021). Furthermore, NMDA receptor channels possess several biophysical properties that are of computational relevance. For instance, through their permeability to Ca^2+^, NMDA-receptor channels are thought to drive synaptic plasticity (Cichon and Gan, 2015; Larkum, 2022). Through their long time-scale, they also are well-suited to modulate feed-forward processing according to context (Iyer et al., 2022; Wybo et al., 2023). Therefore, extending the Ca-AdEx model with distal compartments enabling NMDA-spike generation would be useful to model realistic neuronal I/O relations and plasticity processes. As our Ca-AdEx model is embedded in a general compartmental modeling framework, it is straightforwardly possible to extend the two-compartment description to one where there are additional dendritic subunits that can produce NMDA-spikes. We demonstrate the potential of our approach by deriving the parameters of these subunits from a realistic L5PC morphology (Figure 9B, Hay et al., 2011), using the method based on resistance matrix fits proposed by Wybo et al. (2021). The somatic and Ca-HZ compartment are then respectively equipped with the AdEx and Ca^2+^-spike mechanisms, where we used the same parameters as the two-compartment model (Supplementary Table S1). It speaks to the robustness of our approach that we achieve qualitatively similar behavior as the two-compartmental model, without refitting any of the parameters (Figure 9C). Furthermore, this extended Ca-AdEx model also reproduced the BAC-firing protocol (Figure 9D). We then equip the apical (Figure 9B, orange) and basal (Figure 9B, blue) compartments with excitatory synapses containing both AMPA and NMDA receptor channels, as well as with an inhibitory GABAergic synapse. The latter is stimulated with a fixed Poisson rate of 20 Hz, whereas for the former we scan a range of firing rates: for the apical synapses, we deliver Poisson rates between 0 and 400 Hz in 2 Hz increments, while for the basal synapses Poisson rates between 0 and 200 Hz are probed, in 1 Hz increments (Figure 9F). Simulations with each set of input rates have been run for 2000 ms, and the average output rate is measured by averaging over five such episodes. The dendritic voltage traces exhibit signatures of the non-linear dynamics associated with NMDA- and Ca^2+^-channels (i.e., long up-states, burst firing, etc; see exemplary traces in Figure 9E). Furthermore, the averaged voltage responses in the apical and basal subunits follow the typical sigmoidal response curve (Schiller et al., 2000; Major et al., 2008; Branco et al., 2010; Poirazi et al., 2003; Singh and Zald, 2015) (Figure 9F, insets). These inset plots show the min-max envelope of the averaged voltage, i.e., for the apical voltage response (orange), the minimal values occur for the lowest basal input level, whereas the maximal value occurs for the highest basal input level. That these min-max envelopes are close together and do not substantially affect the sigmoidal response curve, demonstrates that apical and basal areas are mutually independent (Wybo et al., 2019). Finally, the supra-linear Ca^2+^-spike mediated interaction between apical and basal areas is clearly visible in the output firing rates, where a strong increase occurs above a 100 Hz basal and a 100 Hz apical input rate (Figure 9F). We also remark that while the input and output rates seem high when considered as tonic firing rates, it is reasonable to assume that such rates can and do occur transiently, through the coincidence of multiple inputs to the apical and/or basal regions.

## 4 Discussion

Here, we introduce the Ca-AdEx model, designed to capture the essential features of the apical-amplification (AA), apical-drive (AD), and apical-isolation (AI) regimes in cortical pyramidal neurons, with a negligible increment of the computational cost compared to classical point-like neuron models. We anticipate that this advancement will facilitate the development of network models capable of emulating the cognitive functions of awake, NREM, and REM-like cortical states, as well as the learning capabilities associated with brain-state-specific bursting in cortical neurons that detect coincident apical and somatic signals. In addition, we hope this will enable more efficient simulations investigating the impact of dendritic mechanisms on consciousness in line with the Dendritic Integration Theory (Aru et al., 2020b) that some authors propose may be a unifying mechanism that can help bridging different theories of consciousness, toward a more integrative, multi-scale view (Storm et al., 2024). Apical mechanisms play a crucial role in optimally combining internal priors and perceptual evidence within multi-areal, hierarchical, cortical systems featuring lateral, top-down, and bottom-up connections (Larkum, 2013; Phillips et al., 2025). A significant observation is the drastic change in the firing rate of neurons where AA is active. This facilitates both fast recognition and classification as well as learning rates that aligns with the sampling rate of world experiences and are compatible with the typical window of a few tens of milliseconds adopted by STDP models (like Song et al., 2000; Gütig et al., 2003) that derive from well consolidated experimental evidence (Markram et al., 1997; Bi and Poo, 1998). Notably, an even more enhanced firing regime may occur during the AD functional mode, potentially related to the replay and association of experiences during dreaming (Aru et al., 2020a; Capone et al., 2022, 2023b), whereas the suppression of integration of inter-areal signals in apical-isolation mode is suggested to underlie the loss of consciousness during anesthesia and deeper sleep stages (Suzuki and Larkum, 2020; Aru et al., 2020a,b).

Also, we can anticipate that the adoption of few-compartment models will be instrumental for a better modeling of the cognitive function of learning and sleep in other areas, starting from the hippocampus.

Furthermore, it is feasible to formulate, at a high level of abstraction, a compact geometric model capturing the effects produced by the combination of signals that convey information about priors and perceptual evidence, segregated into the apical and somatic compartments. The transfer function of the two-compartment Ca-AdEx model described here can be approximated piecewise by low-order polynomials. Specifically, we examined the case of two approximating planes, giving rise to a class of transfer functions we have named ThetaPlanes (*I*_*s*_, *I*_*d*_). ThetaPlanes represents a generalization for two-compartment neurons of the ReLU function commonly used to approximate single-compartment neuron models in numerous artificial intelligence algorithms. ThetaPlanes transfer functions can be implemented as efficient computational gates for use in large cognitive networks at a high level of abstraction. In future works, we plan to investigate the benefits of this computational gate in next-generation bio-inspired artificial intelligence algorithms. In our view, ThetaPlanes could influence multiple lines of development. During learning, a contextual drive applied to a subset of connections may reduce the risk of forgetting previously acquired knowledge, while simultaneously supporting the integration of synergistic activity from neurons not directly targeted by the contextual stimulus. This may also help reduce the number of required training epochs. As one moves up the hierarchy of a layered network, contextual signals are expected to shift from conveying unsupervised information to supporting more supervised or categorical knowledge. During inference, priors conveyed by contextual signals could help reduce classification errors. Proper use of contextual signals may also enhance the network's explainability by labeling meaningful subsets of nodes. This expectation is supported by recent studies about the utility of brain-state-specific bursting regimes demonstrated in Capone et al. (2023b, 2022). These studies, while assuming the existence of such coincidence detection mechanisms as working hypotheses, lacked a biologically grounded transfer function.

Furthermore, the potential to maintain compatibility with the transfer function of widely adopted leaky integrate-and-fire models with adaptation, when AA is not triggered, is promising. In our case, we aimed for compatibility with the Adaptive Exponential Integrate-and-Fire model (AdEx), which is extensively used for simulations at both micro- and meso-scales. It also serves as the basis for mean-field models for simulations encompassing the whole cortex (Capone et al., 2023a). We anticipate that during wakefulness, apical mechanisms and the sparsity of long-range connections will place a strict minority of neurons in a bursting regime. This adjustment is unlikely to significantly alter the average spectral signatures of expressed rhythms but could induce profound effects on perception and learning ability. Such a balance is necessary to maintain compatibility with the extensive body of experimental evidence concerning rhythms, average firing rates, and their fluctuations. During sleep, we anticipate that a delicate balance will be maintained to ensure healthy sleep patterns and to promote its beneficial cognitive and energetic effects.

An additional noteworthy observation is that two-compartment neurons with significant transfer functions have been efficiently discovered using the L2L framework within the majority of replicas of simulations of an evolutionary process that spanned only a hundred generations, each including no more than a hundred individuals.

In our view, this suggests that natural evolution could have readily identified the cognitive advantages of apical mechanisms through localized variations of membrane and channel parameters, in ways somewhat analogous to the creation of two compartments. Thus, evolution might have incrementally given rise to the complex morphology seen in pyramidal neurons in the cortex.

Another aspect touched by our work is the role of high-performance computing (HPC) infrastructure, which offers a platform for conducting increasingly robust, comprehensive, and extensive explorations of parameter spaces in scientific models. Coupled with machine learning, HPC emerges as a potent digital environment for adaptive testing and understanding the interactions between data and models. HPC allows scientists to simultaneously test a vast number of hypotheses within short time frames, delivering crucial information that can be incorporated into accelerated experimental cycles. Within this framework, L2L serves as an accessible tool for domain scientists to interface with HPC and conduct efficient parameter explorations. It allows focusing on areas of interest while offering a comprehensive overview of the entire parameter space, including the relationships between parameters and the selected fitness metrics. In this manuscript, we demonstrated that L2L is a framework adept at leveraging HPC infrastructure to assist neuroscientists in optimizing, fitting, and searching for suitable dynamics in models. Specifically, following the definition of the genome and the fitness functions for the multi-compartment neuron, an evolutionary algorithm can identify suitable candidates that survive the selection process.

This work, based on a customization of the multi-compartment framework available in NEST (Gewaltig and Diesmann, 2007; Spreizer et al., 2022), also facilitates the inclusion of two- and many-compartments neuron models supporting apical mechanisms in the ecosystem of other standard simulation engines like Neuron (Carnevale and Hines, 2006), Brian (Stimberg et al., 2019) and Arbor (Abi Akar et al., 2019). This work also outlines an approach grounded in traditional compartmental dynamics, which is computationally efficient and accurately captures the interplay between somatic action potentials (APs) and dendritic Ca2+-spikes. As part of a broader compartmental modeling framework in NEST, our model can easily be expanded with additional compartments to represent other dendritic events, such as N-methyl-D-Aspartate (NMDA) spikes. Finally, due to its implementation in NEST, the model can be directly integrated into network simulations modeling incremental learning and sleep cycles (Capone et al., 2019; Golosio et al., 2021).

## Data Availability

The parameters of the neuron model presented in the study, the list of fitness functions and other details are included in the Supplementary material, further inquiries can be directed to the corresponding author(s).
