# Supplementary material for: Simplified two-compartment neuron with calcium dynamics capturing brain-state specific apical-amplification, -isolation and -drive
Source: Front Comput Neurosci. 2025 May 20;19:1566196. doi: 10.3389/fncom.2025.1566196 (PMC12130020; doi:10.3389/fncom.2025.1566196)
Supplement: Supplementary file 1 [file Data_Sheet_1.pdf]

Supplementary Material

1 PARAMETERS OF THE CA-ADEX NEURON MODEL

Table S1 lists the full *genome* of the Ca-AdEx neuron model. The values of the whole set of parameters are those identified by the evolutionary search, as described in the Materials and Methods section of the paper.

Table S1. Neuron *genome*: parameters characterizing the Ca-AdEx neuron identified by the evolutionary search algorithm.

**Soma parameters**			
C_m^s	Membrane capacitance	246.7882968598874	pF
g_L^s	Leakage conductance	5.0	nS
E_L^s	Leakage reversal potential	-69.24596493128396	mV
t_{ref}	Refractory period	0	ms
Δ_T	Slope factor	2	mV
a	Subthreshold adaptation	0	nS
b	Spike-triggered adaptation	40	pA
τ_w	Adaptation time constant	500	ms
g_w	Adaptation coupling conductance	1.1156385639067352	nS
V_{th}	Membrane voltage threshold v	-50	mV
V_{reset}	Membrane voltage after-spike reset	-61.73952230767877	mV
w_{BAP}	BAP amplitude	27.995561755479308	mV
d_{BAP}	BAP delay	0.1195980511869619	ms
**Distal parameters**			
C_m^d	Membrane capacitance	23.67372778891213	pF
g_L^d	Leakage conductance	3.377855016658499	nS
E_L^d	Leakage reversal potential	-55.000000000000014	mV
g_C	Soma-distal coupling conductance	19.777320239615996	nS
$\bar{g}_{Ca}$	Ca current maximal conductance	21.045506331690845	nS
τ_{Ca}	Ca current decay time constant	103.57233790866408	ms
τ_m	Ca current activating function time constant	15	ms
τ_h	Ca current deactivating function time constant	80	ms
m_{half}	Ca current activating function half voltage	-9	mV
h_{half}	Ca current deactivating function half voltage	-21	mV
m_{slope}	Ca current activating function slope	0.5	-
h_{slope}	Ca current deactivating function slope	-0.5	-
$[Ca]_{th}$	Ca concentration threshold for Ca channel opening	0.00043	mM
$[Ca]_0$	Baseline intra-cellular Ca concentration	0.0001	mM
ϕ	Scaling factor in Ca concentration dynamics	3.9283098522841e-08	-
$\bar{g}_{K_{Ca}}$	Ca dependent K current maximal conductance	13.199867205029523	nS
$\tau_{K_{Ca}}$	Ca dependent K current activating function time constant	1	ms
$const_{K_{Ca}}$	Exponential factor in Ca dependent K current	4.8	-
E_K	K reversal potential	-90	mV

2 PARAMETERS OF THE REFERENCE ADEX NEURON MODEL

Table S2 contains the parameters of the AdEx point-like neuron model used as a reference for the behaviour of the Ca-AdEx model when stimulated with pure somatic input current.

Table S2. Neuron *genome*: parameters characterizing the Ca-AdEx neuron identified by the evolutionary search algorithm.

C_m^s	Membrane capacitance	200	pF
g_L^s	Leakage conductance	10	nS
E_L^s	Leakage reversal potential	-63	mV
t_{ref}	Refractory period	0	ms
Δ_T	Slope factor	2	mV
a	Subthreshold adaptation	0	nS
b	Spike-triggered adaptation	40	pA
τ_w	Adaptation time constant	500	ms
V_{th}	Membrane voltage threshold v	-50	mV
V_{reset}	Membrane voltage after-spike reset	-65	mV

3 FITNESS FUNCTION

Table S3 lists the fitness functions used to guide the parameter search, trough the optimization of the *pulse stimuli* and *prolonged stimuli* tasks. Note that the target of the optimization is to bring towards zero a set of squared losses, and the total $L2$ is the sum of the set of individual $L2\_SPECIFIC$ reported in the table.

Table S3. Fitness functions

**Pulse stimuli task**	
$L2\_PG$	Diff. of spikes between (I_s th, I_d under-th) and ($I_s = 0, I_d$ over-th) [target=1]
$L2\_PE$	Number of spikes for ($I_s = 0, I_d$ over-th) [target=2]
$L2\_PD$	Number of spikes for (I_s th, I_d under-th) [target=3]
$L2\_PR$	I_d over-th / I_d under-th ratio
**Prolonged stimuli task**	
Primary checks of apical channels activation	
$L2\_CaO$	Missed Ca channel opening
$L2\_CaC$	Missed closure of apical mechanism
$L2\_CaO\_soma$	Check for Ca NOT opening for $I_d = 0$
$L2\_CaH$	Ca opening threshold
AdEx matching	
$L2\_SEMD$	EMD between firing of AdEx and Ca-AdEx for ($I_d = 0$)
$L2\_SH$	Rheobase for ($I_d = 0$)
$L2\_LFR$	Firing value at largest explored I_s for ($I_d = 0$)
$L2\_CVISIS$	Check for irregular spiking on somatic input
$L2\_CMS$	Soma capacitance
Gain & linearity of apical mechanism	
$L2\_120CaH$	Distal firing rate after Ca opening
$L2\_LinJump$	Linearity in Ca jump for growing (Is,Id) currents
$L2\_Monoton$	Monotonicity of the f/I curves
$L2\_NumJump$	Number of jumps in f/I curves
Exclusion of pathological configurations	
$L2\_E$	Check for epileptic neuron
Cautionary checks	
$L2\_MINV$	Minimum voltage
$L2\_DH$	Rheobase for ($I_s = 0$)
$L2\_JACaO$	Check for multiple jumps in firing rate after calcium opening

4 PARAMETERS OF THE *THETAPLANES* FUNCTION

Table S4 contains the parameters that define the *ThetaPlanes* function and their values for the fitted configuration: the $\upsilon_-(I_s, I_d)$ and $\upsilon_+(I_s, I_d)$ planes, the transition line to high firing rates, and the rheobase.

Table S4. Parameters of the *ThetaPlanes* piece-wise linear approximating function. See Supplementary Material for representative values.

ν_+ **plane**			
a_+	apical amplification region	0.048	Hz/pA
b_+		0.005	Hz/pA
d_+		29.367	Hz/pA
ν_- **plane**			
a_-	lower firing rate region	0.048	Hz/pA
b_-		0.027	Hz/pA
d_-		-1.432	Hz/pA
$I^H_{d,F}(I_s)$			
θ^H_m	separation between regions	-0.266	-
θ^H_q		549.397	pA
$I^\rho_{d,F}(I_s)$			
θ^ρ_m	rheobase line	-1.754	-
θ^ρ_q		51.053	pA

5 PARAMETER SPACE EXPLORATION WITH L2L

As mentioned in section ***2.4 The Learning to Learn framework*** in the main manuscript, the parameter exploration was performed using the L2L framework. L2L is equipped to iteratively deploy instances of the inner loop on HPC resources in a variety of ways. It is compatible with any scheduler present on a cluster or supercomputer. In this work, the *SLURM Workload Manager* served as the scheduler to allocate the necessary computational resources. The parameter space exploration has been executed on two computing platforms: 1) on the JUSUF supercomputer at the Jülich Supercomputing Centre (136 standard compute nodes 2× AMD EPYC 7742, 2× 64 cores, 2.25 GHz, 256 (16× 16) GB DDR4, 3200 MHz, InfiniBand HDR100 (Connect-X6), Rocky Linux 8 distribution) and, 2) on a partition of the local cluster available at the INFN APElab, Sezione di Roma (6 nodes 2x AMD EPYC 7313 16-Core Processor, 3.0 GHz, 128 GB, Centos 7 distribution).

Hereafter, an example of the L2L execution script used in this work.

```
#
# CaAdEx main code for genetic algorithm optimization
#
# First version: 26/09/2024
# Author: Elena Pastorelli, INFN, Rome (IT)

import os
import yaml
```

```
import numpy as np
import pickle

from datetime import datetime
from random import randint
from l2l.utils.experiment import Experiment

from l2l.optimizers.evolution import GeneticAlgorithmParameters
from l2l.optimizers.evolution import GeneticAlgorithmOptimizer
from l2l.optimizees.CaAdEx1 import CaAdExOptimizee
from l2l.optimizees.CaAdEx1 import CaAdExOptimizeeParameters

def run_experiment():

    timestamp = datetime.now().strftime("%Y-%m-%d-%H_%M_%S")
    randval = str(randint(0,1000))
    filename = 'CaAdEx1_JU' + timestamp + randval

    # Sending the experiment results to the SCRATCH partition
    experiment = Experiment(root_dir_path='/p/scratch/test')

    runner_params = {
        "srun": "srun -n 1 -c 1 --exact",
        "exec": "python3",
        "max_workers": 100
    }
    traj, _ = experiment.prepare_experiment(
            runner_params=runner_params,
            name=filename,
            log_stdout=True,
            debug=True,
            stop_run=True,
            overwrite=True)

    # Optimizee params
    optimizee_parameters = CaAdExOptimizeeParameters()
    optimizee = CaAdExOptimizee(traj, optimizee_parameters)

    optimizer_seed = 1234
    optimizer_parameters = GeneticAlgorithmParameters(seed=26,
                                                      pop_size=100,
                                                      cx_prob=0.5,
                                                      mut_prob=0.5,
                                                      n_iteration=100,
                                                      ind_prob=0.3,
```

```
                                                     tourn_size=7,
                                                     mate_par=0.5,
                                                     mut_par=0.5)

    optimizer = GeneticAlgorithmOptimizer(traj,
        optimizee_create_individual=optimizee.create_individual,
        parameters=optimizer_parameters,
        optimizee_bounding_func=optimizee.bounding_func)
    # Run experiment
    experiment.run_experiment(
        optimizer=optimizer, optimizee=optimizee,
        optimizer_parameters=optimizer_parameters,
        optimizee_parameters=optimizee_parameters)
    # End experiment
    experiment.end_experiment(optimizer)

def main():
    run_experiment()

if __name__ == '__main__':
    main()
```

In this work, for each optimization run, we have requested a single allocation which comprises enough computational resources to launch all individuals in each iteration of the outer loop. In our case the allocation is obtained through a SLURM `sbatch` script that typically allocates a total number of cores greater than the number of individuals in each iteration because the simulation of each two-compartment neuron can fit on a single core. Subsequently, for each iteration, L2L launches a number of steps equal to the number of individuals. This distribution is achieved by setting the appropriate scheduler parameters. In L2L this means defining the required resources per individual within the `srun` entry in the `runner_parameters` configuration. An example of such an entry is:

```
    runner_params = {
        "srun": "srun -n 1 -c 1 --exact",
        "exec": "python3",
        "max_workers": 100}
```

where the key `srun` is the entry to configure a SLURM step within an existing allocation (can be left empty in case of a local run in a system without SLURM or set to another scheduler), the `exec` key defines the executable for the optimizee (in this case python) and `max_workers` defines the number of computing instances deployed in the computer (in this case 100). The number of workers can be less than the number of individuals of each iteration but this would result in serializing the execution inside the single iteration. The user defines the parameters for the parallel execution of the individuals using the `srun` key. In this case the associated value instructs to use MPI encapsulated by the `srun` command: the parameter `-n` defines the number of MPI processes, `-c` denotes the number of cores per process assigned to each individual and `--exact` tells the scheduler to assign only the previously specified resources to each individual within this SLURM step. An additional parameter `-N` can be used to specify the number of nodes.

The optimization used in this work is based on a genetic algorithms defined by a set of parameters as detailed in the script. In particular, the `optimizer_parameters` are set for the Blend Crossover operation, the Gaussian mutation algorithms and for the Tournament selection performed to as part of the evolutionary optimization.

L2L also allows the end user to restart optimization or parameter exploration runs from a previous run using a checkpointing functionality. This feature allows end users to continue simulations which were stoped due to time or resource limitations. It also allows users to change parameters in the outer loop to tune the exploration based on previous partial results.

The version of L2L used in this work can be accessed here: https://github.com/Meta-optimization/L2L.git in the brain `replace_jube`.

6 ERRORS IN FITTING THE TRANSFER FUNCTION

Figure S1A and **S1B** show the error (in Hz) between the planar fits $\nu_{-,+}(I_s, I_d)$ and the simulated transfer functions $\nu(I_s, I_d)$. The discrepancy between the firing rates obtained from simulation of the two-compartment Ca-AdEx neuron and those predicted by the fitting plane is discretized into 0.5 Hz intervals, reflecting that firing rates are measured over simulation periods lasting two seconds.

Figure S1C illustrates the linear fit of the data representing the boundary between M_+ and M_-. This linear fit leads to the definition of the parameters θ_m^H, the slope of the fitting line, and θ_q^H, its offset, as detailed in the main article text.

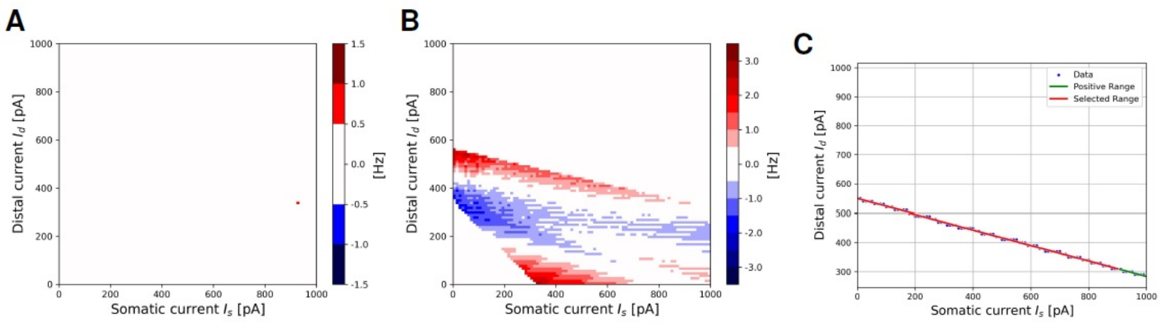

Figure S1. ThetaPlanes analysis. (A) Errors of fitting planes in M_+ region: $\nu_+ - \nu$ (Hz). **(B)** Errors of fitting planes in M_- region: $\nu_- - \nu$ (Hz). **(C)** Linearity of the separation between the high activity M_+ region and the low activity M_- region. The red line represent the linear fit $I_{d,F}^H(I_s)$.
